# Hypoxia-Induced Intracellular and Extracellular Heat Shock Protein gp96 Increases Paclitaxel-Resistance and Facilitates Immune Evasion in Breast Cancer

**DOI:** 10.3389/fonc.2021.784777

**Published:** 2021-12-20

**Authors:** Tian Tian, Jiguang Han, Jian Huang, Shangziyan Li, Hui Pang

**Affiliations:** ^1^ Department of Oncology, Harbin Medical University Cancer Hospital, Harbin, China; ^2^ Department of Surgical Oncology, Harbin Medical University Cancer Hospital, Harbin, China

**Keywords:** heat shock protein gp96, p53, breast cancer, cancer immunology, paclitaxel-resistance

## Abstract

**Backgrounds:**

Hypoxia contributes to cancer progression, drug resistance and immune evasion in various cancers, including breast cancer (BC), but the molecular mechanisms have not been fully studied. Thus, the present study aimed to investigate this issue.

**Methods:**

The paclitaxel-sensitive BC (PS-BC) cells were administered with continuous low-dose paclitaxel treatment to establish paclitaxel-resistant BC (PR-BC) cells. Exosomes were isolated/purified by using the commercial kit, which were observed by Transmission electron microscopy (TEM). Cell viability was measured by MTT assay, cell apoptosis was determined by flow cytometer (FCM). Gene expressions were respectively measured by Real-Time qPCR, Western Blot and immunofluorescence staining assay. The peripheral mononuclear cells (PBMCs) derived CD8^+^ T cells were obtained and co-cultured with gp96-containing exosomes, and cell proliferation was evaluated by EdU assay. ELISA was employed to measure cytokine secretion in CD8^+^ T cells’ supernatants.

**Results:**

HSP gp96 was significantly upregulated in the cancer tissues and plasma exosomes collected from BC patients with paclitaxel-resistant properties. Also, continuous low-dose paclitaxel treatment increased gp96 levels in the descendent PR-BC cells and their exosomes, in contrast with the parental PS-BC cells. Upregulation of gp96 increased paclitaxel-resistance in PS-BC cells *via* degrading p53, while gp96 silence sensitized PR-BC cells to paclitaxel treatments. Moreover, PR-BC derived gp96 exosomes promoted paclitaxel-resistance in PS-BC cells and induced pyroptotic cell death in the CD8^+^ T cells isolated from human peripheral blood mononuclear cells (pPBMCs). Furthermore, we noticed that hypoxia promoted gp96 generation and secretion through upregulating hypoxia-inducible factor 1 (HIF-1), and hypoxia increased paclitaxel-resistance and accelerated epithelial-mesenchymal transition (EMT) in PS-BC cells.

**Conclusions:**

Hypoxia induced upregulation of intracellular and extracellular gp96, which further degraded p53 to increase paclitaxel-sensitivity in BC cells and activated cell pyroptosis in CD8^+^ T cells to impair immune surveillance.

## Backgrounds

Paclitaxel-resistance is a huge obstacle for the treatment of breast cancer (BC), which makes this chemical drug ineffective for the treatment of BC, resulting in worse prognosis and recurrence in BC patients (1, 2). Although researchers have used paclitaxel-other chemical drugs combined therapy to treat BC, the therapeutic efficacy of this treatment strategies is still seriously limited as the results of its unclear molecular mechanisms (3–5). Thus, it becomes necessary and meaningful to delve into the molecular mechanisms by which BC cells transform from paclitaxel-sensitive BC (PS-BC) cells into the paclitaxel-resistant BC (PR-BC) cells. In recent studies, it draws our attention that cancer cells derived exosomes play critical role in regulating paclitaxel-resistance in BC (6, 7). Specifically, data from Pederson et al. show that paclitaxel treatment is able to alter the secretion patterns of extracellular vesicles in BC cells (6), and Yang et al. further evidence that chemotherapy-elicited microRNAs (miRNAs)-containing exosomes promote BC cell stemness and chemoresistance (7). Heat shock protein (HSP) glucoprotein 96 (gp96) is reported as the components of cancer derived exosomes (8, 9), and gp96 itself is related with the aggressiveness of BC (10, 11) and paclitaxel-resistance in ovarian cancer cells (12). However, it is still not reported whether gp96-containing exosomes involve in regulating paclitaxel-resistance in BC.

Hypoxia is a typical feature in solid tumors when tumor volume reaches about 2 mm^3^, and the inner cancer cells suffer from oxygen and nutrients deprivation (13–15). Under those environmental stresses, the expression patterns of various cancer-associated genes are altered, and a subgroup of the cancer cells acquire the tumor-promoting properties to facilitate angiogenesis and cancer metastasis (16–18). Interestingly, recent studies also evidence that hypoxia influences chemo-resistance in multiple cancers, such as glioma (19), hepatocellular carcinoma (20), and BC (21). Of note, Tang et al. report that hypoxia promotes BC cell growth *via* modulating glycogen metabolic program (22), and data from Wu et al. suggest that hypoxia facilitates metastasis and chemoresistance in BC (21). To our knowledge, hypoxia is capable of inducing the upregulation of HSPs to initiate the protective mechanisms in the cells under hypoxic stresses (23–25), and Kutomi et al. evidence that hypoxia upregulates gp96 levels (26). In addition, hypoxia-inducible factor 1 (HIF-1) is the key hypoxia-associated gene, which is reported to regulate BC progression (27–29) and paclitaxel-resistance (21, 30). Interestingly, as previously reported, production of gp96 is coordinately induced by heat shock factor 1 (HSF-1) and HIF-1 (9), and both HIF-1 and gp96 are elevated in malignant mesothelioma (31).

To our knowledge, human immune system initially eliminates cancer cells through recognizing tumor associated antigens, which is known as immune surveillance (32–34), and the main tumor killer cells are considered as CD8^+^ T lymphocytes (35, 36). However, cancer cells develop strategies to hide tumor antigens or induce immune cell death to impair the anti-tumor functions of the immune system, and this process is known as immune evasion (35, 36). According to the previous literatures (37–39), cancer cells derived exosomes are pivotal to induce immune cell death, and Hong et al. report that non-small cell lung cancer (NSCLC) derived PD-L1 containing exosomes promote CD8^+^ T cell death (35). Interestingly, gp96 is reported to be closely associated with immune cells infiltration in BC (9, 40). Researchers notice that gp96 affects CD4^+^ and CD8^+^ T-lymphocytes in various types of invasive breast carcinoma (40), and extracellular gp96 has been considered as biomarkers for immune surveillance and immune evasion (9). Moreover, recent data suggest that there exist links between hypoxia and immune evasion (41), and hypoxia-induced extracellular HSPs influences immune surveillance and immune evasion (9).

Based on all the existed information, the present study was designed to mainly investigate the three academic issues as follows: (1) The role of gp96 in regulating paclitaxel-resistance in BC, and uncover the potential mechanisms. (2) The mechanisms by which gp96-containing exosomes facilitate immune evasion in BC. (3) The involvement of hypoxia in regulating paclitaxel-resistance and EMT in BC.

## Materials and Methods

### Clinical Specimen Collection

BC patients (N = 67) were recruited in the Harbin Medical University Cancer Hospital from 2016 to 2020, and the cancerous and non-cancerous tissues were collected by surgery, were judged by two experienced pathologists in our hospital, and were immediately stored at -80°C conditions for further utilization. According to their therapy treatment history, the BC patients were divided into two groups with (N = 34, considered as PR-BC patients) or without (N = 33, considered as PS-BC patients) paclitaxel treatment history. Specifically, the patients who had paclitaxel treatment history were considered as PR-BC patients, whereas the patients did not receive any chemotherapy were deemed as PS-BC patients. The clinical characteristics of the involved patients were listed in **Table 1**. The clinical experiments were approved by the Ethics Committee Affiliated to Harbin Medical University Cancer Hospital, and the informed consent forms were also signed by all the participates.

**Table 1:** Correlations of gp96 mRNA with the clinical parameters in BC patients.

	Cases (N)	mRNA levels of gp96		*P* values
		Low	High	
Age (Years)				0.432
≤40	23	11	12	
>40	44	31	13	
Clinical stage				**0.012**
I/II	41	21	20	
III/IV	26	12	14	
Lymph node metastasis				**0.001**
Negative	18	10	8	
Positive	49	23	26	
Tumor size (cm)				**<0.0001**
≤4	29	19	10	
>4	38	15	23	

The bold values means that the data was statistical significance.

### Cell Culture, Treatment and Vectors Transfection

The human TNBC cell lines (MDA-MB-453 and MCF7) were purchased from BeNa Culture Collection (Beijing, China), All cells were regularly authenticated using short tandem repeat (STR) DNA profiling and tested to confirm that they were mycoplasma contamination-free. The above cells were maintained in the DMEM medium (Gibco, USA) containing 10% fetal bovine serum (FBS, Gibco) under the conditions with 5% CO_2_ atmosphere at 37°C. In addition, according to the experimental protocols recorded in our previous work (42), the paclitaxel-resistant MDA-MB-453/PTX and MCF7/PTX cell lines was established by treating the parental paclitaxel-sensitive BC cells with continuous paclitaxel treatment in a step-wise manner. The PR-BC cells were maintained in the DMEM medium (Gibco, USA) containing 10% fetal bovine serum, and the PR-BC cells at passage 2 to 4 with paclitaxel-resistant properties were used in this study. Moreover, the vectors for gp96 overexpression/downregulation and p53 overexpression were synthesized by Sangon Biotech (Shanghai, China), which were delivered into the TNBC cells by using the Lipofectamine 2000 transfection reagent (Invitrogen, USA). Moreover, the PS-BC cells were exposed to the hypoxic environment (0.2% oxygen, 5% CO_2_, and 37°C) for three days to establish the hypoxic PS-BC cell models as previously described (43).

### Isolation and Purification of the Exosomes

The EXO Quick-Exosome Isolation kit (Biosciences System, USA) was purchased to obtain the exosomes from patients’ plasma and cells’ supernatants in keeping with the manufacturer’s protocols. Specifically, for the plasma exosomes, the patient blood was collected and were subjected to centrifugation (3000g, 15min) to acquire the supernatants, which were regarded as plasma. Then, 300 μl of the plasma were mixed with the exosome precipitation solution of the kit, and the plasma exosomes were further collected. For the cell-derived exosomes, the BC cells were cultured *in vitro* and their supernatants were collected through sequential centrifugation, including 300g for 10 min, 2000g for 15min, 12,000g for 30 min to remove the cells and debris, which were further filtered by 0.22μm filters (Millipore, USA). The filtered supernatants were mixed with the working solution of the kit, and the exosomes were isolated and purified according to the kit’s instructions.

### Transmission Electron Microscopy (TEM)

To observe the morphologies of the exosomes, we performed the TEM assay based on the previous protocols. The purified exosomes were fixed with 1% glutaraldehyde for 10 min at room temperature, and the exosomes were further placed onto a formvar carbon-coated 300-mesh copper electron microscopy grids (Agar Scientific Ltd., USA) for 5 min, and the exosomes were stained with 2% uranyl oxalate for 2 min. The grids were washed with PBS and a TEM machine (Jeol, Japan) was employed to observe and photograph the exosomes.

### Preparation of the CD8^+^ T Cells

The CD8^+^ T cells were isolated and cultured according to the previous literature (35). Briefly, the volunteers were recruited and the blood was obtained, which were further subjected to the Ficoll kit (Sigma-Aldrich, USA) to obtain the peripheral mononuclear cells (PBMCs) in keeping with the reagent’s protocols. A flow cytometer (BD Bioscience, USA) was further used to acquire the CD8^+^ T subgroup from the PBMCs as previously described (44).

### Real-Time qPCR

Total RNA was extracted from BC tissues and cells through using the TRIzol reagent (Beyotime, Shanghai, China), and further Real-Time qPCR was performed to quantify genes expressions. Specifically, the cDNA synthesis kit (ThermoFisher Scientific, CA, USA) was used to reversely transcribed the mRNA into complementary DNA (cDNA), through using the Bio-Rad Real-Time qPCR detection system (Bio-Rad, CA, USA), we performed Real-Time qPCR to determine the mRNA levels of HSP gp96, p53, IL-1β, IL-18, ZEB1, Vimentin, Twist, Slug and Snail, and the relevant expression levels of the above genes were normalized by the GAPDH. The primers were designed and constructed by Sangon Biotech (Shanghai, China), and their detailed sequences were shown in **Table 2**.

**Table 2:** The primer sequences for Real-Time qPCR.

Gene name	Primer sequences
HSP gp96	Forward: 5′-GCT TCG GTC AGG GTA TCT TTT-3′
	Reverse: 5′-CAC CTT TGC ATC AGG GTC AAT-3′
GAPDH	Forward 5′-TGT TGC CAT CAA TGA CCC CTT-3′
	Reverse 5′-CTC CAC GAC GTA CTC AGC G-3′
p53	Forward: 5’-GAC GGT GAC ACG CTT CCC TG-3’
	Reverse: 5’-CAC CAC CAC ACT ATG TCG-3’
IL-1β	Forward: 5’-GGC AGG TGG TAT CGA TCA TC-3’
	Reverse: 5’-CAC CTT GGA TTT GAC TTC TA-3’
IL-18	Forward: 5’-GCT GGC TGT AAC CCT CTC TG-3’
	Reverse: 5’-TTC CTC CTT TTG GCA AGC TA-3’
ZEB1	Forward: 5’-GCT GGC AAG ACA ACG TGA AAG-3’
	Reverse: 5’-GCC TCA GGA TAA ATG ACG GC-3’
Vimentin	Forward: 5’-CGT CCA CAC GCA CCT ACA G-3’
	Reverse: 5’-GGG GGA TGA GGA ATA GAG GCT-3’
Twist	Forward: 5’-GGA CAA GCT GAG CAA GAT TCA-3’
	Reverse: 5’-CGG AGA AGG CGT AGC TGA G-3’
Slug	Forward: 5’-TGG TCA AGA AAC ATT TCA ACG CC-3’
	Reverse: 5’-GGT GAG GAT CTC TGG TTT TGG TA-3’
Snail	Forward: 5’-CAC ACG CTG CCT TGT GTC T-3’
	Reverse: 5’-GGT CAG CAA AAG CAC GGT T-3’
Fibronectin	Forward: 5’-ATG TGG ACC CCT CCT GAT AGT-3’
	Reverse: 5’-GCC CAG TGA TTT CAG CAA AGG-3’

### Western Blot Analysis

RIPA lysis buffer (Biotime, Hangzhou, China) was used to obtain total proteins from BC tissues and cells, and the quality of the protein was determined by BCA kit (Pierce, USA). Proteins were separated by 10% SDS-PAGE, and were subsequentially transferred onto the PVDF membranes (Millipore, MA, USA). The membranes were blocked by 5% non-fat milk, and incubated with the primary antibodies against gp96 (1:1000, #ab227293, Abcam, UK), TSG101 (1:1000, #ab125011, Abcam, UK), p53 (1:2000, #ab1101, Abcam, UK), GAPDH (1:2000, #ab8245, Abcam, UK), CD63 (1:1500, #ab271286, Abcam, UK) and β-actin (1:2000, #ab179467, Abcam, UK) at 4°C overnight. In the second day, the membranes were probed with the secondary antibodies for 1h at room temperature, and an ECL system (Bio-Rad, CA, USA) was employed to visualize the protein bands. The expression levels of the proteins were normalized by GAPDH.

### MTT Assay

MTT assay was performed to evaluate cell viability as previously reported. In brief, the BC cells were cultured in the 96-well plates in the standard culture conditions, which were further added with MTT reaction solution at the density of 20 μl per well for 4h. Then, the supernatants were carefully discarded, and the formazan was diluted by adding 150 μl DMSO. The plates were fully vortexed, and a microplate reader (Molecular Devices, CA, USA) was employed to determine cell viability at the wavelength of 450 nm.

### Enzyme Linked Immunosorbent Assay (ELISA)

The cytokine secretion (IL-1β and IL-18) in BC cells’ supernatants were determined by performing ELISA through using their corresponding ELISA kit purchased from RAPIDBIO (CA, USA). The cell supernatants were collected and incubated with the reaction solution, and a microplate reader (Molecular Devices, CA, USA) was further employed to measure the optical density (OD) values at the wavelength of 450 nm, and the relative expression levels of the two cytokines were calculated according to the OD values.

### Measurement of Cell Apoptosis by Flow Cytometry (FCM)

The BC cells with differential treatments and vector transfection were sequentially stained with Annexin V-FITC and PI in the commercial apoptosis detection kit (Solarbio, Beijing, China), and a FCM (BD Bioscience, USA) was employed to examine the apoptotic cell ratio, which were defined as Annexin V-FITC or PI-positive cells. The experiments were conducted in accordance with the manufacturer’s instruction.

### EdU Assay

The EdU kit (Solarbio, Beijing, China) was purchased to examine cell proliferation in keeping with the manufacturer’s protocol. Specifically, the CD8^+^ T cells with or without gp96-containing exosomes co-incubation were incubated with EdU reaction solution for 2h, and the cells were further washed by PBS buffer for three times, fixed by 4% paraformaldehyde, neutralized by glycine, and reacted with fluorescence dyes. Then, the cells were stained with DAPI to visualize their nucleus, and a fluorescence microscope (Olympus, Japan) was employed to observe and photograph the EdU-positive cells, which were regarded as CD8^+^ T cells with good proliferative abilities.

### Immunofluorescence Staining Assay

The BC cells were cultured in the 25 cm^3^ flasks and were fixed by incubating cells with 4% paraformaldehyde for 20 min at 37°C. Then, the fixed cells were washed by PBS buffer for three times to fully remove the residual paraformaldehyde, and were permeabilized by using the PBST buffer (PBS buffer with 0.05% Triton X-100 supplementation). Then, the cells were sequentially incubated with the primary antibodies (Gasdermin D, 1:200) and secondary antibodies, and the expression levels and cellular localization of the proteins were examined by performing a confocal microscope (Zeiss, Germany).

### Animal Experiments

The MCF7 cells were subcutaneously injected into the dorsal flank of the six-week-old female C57BL/6J mice, and the cell concentration for injection was 1 × 10^6^ cells per mouse, and tumor volume was monitored every 3 days. Until the tumor volume reached about 100 mm^3^, the gp96-containing or deficient exosomes were injected into the mice tumors, and the tumor volume were further monitored for 6 days with 2 days intervals. The mice were divided into three groups with 6 mice each group, including negative control (NC), gp-96 containing exosomes, and gp-96-deficient exosomes. Then, the mice were anesthetized and sacrificed, and the tumors were obtained and weighed. Our animal experiments were conducted in accordance with the guidelines of the Ethics Committee affiliated to Harbin Medical University Cancer Hospital.

### Analysis of the Data

SPSS 18.0 software was employed to analyze the data in the present study, which had been shown as Means ± Standard Deviation (SD), and the graphs were generated by using the GraphPad Prism 8.0 software. One-way ANOVA analyzed the differences among multiple groups (>2), while the differences in two groups were compared by performing the student’s t-test. Individual experiment was repeated at least 3 times. *P < 0.05, **P < 0.01 and ***P < 0.001 were considered as statistical significance.

## Results

### Intracellular and Extracellular gp96 Was Closely Related With Cancer Malignancy and Paclitaxel-Resistance in BC

**Figure 1:**
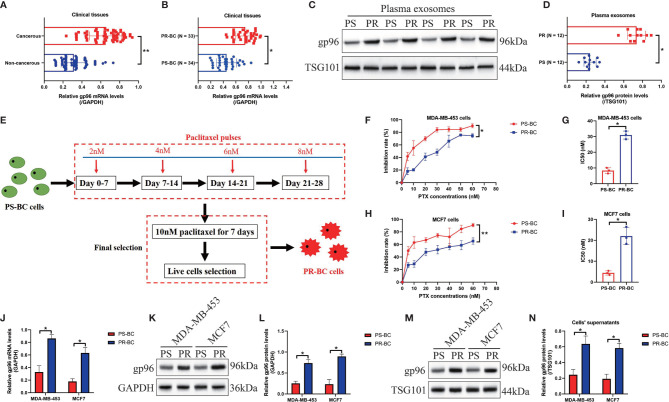
Intracellular and extracellular gp96 was relevant to cancer progression and paclitaxel-resistance in BC. **(A, B)** The mRNA levels of gp96 in the clinical BC tissues were analyzed by Real-Time qPCR. **(C, D)** The gp96 protein levels in BC patients with or without paclitaxel-resistant properties were determined by Western Blot analysis. **(E)** Experimental procedures for the establishment of PR-BC cells. **(F–I)** The BC cells were exposed to differential doses of paclitaxel for 48h, and the inhibition rate and IC50 were determined by MTT assay. The **(J)** mRNA and **(K, L)** protein levels of gp96 in BC cells and their supernatants were respectively examined. **(M, N)** The exosomes in the BC cells’ supernatants were collected, and the expression levels of exosomal gp96 were examined by Western Blot analysis, which were normalized by exosome marker TSG101. *P < 0.05; **P < 0.01.

For the clinical experiments, the cancerous and adjacent non-cancerous tissues were collected from BC patients (N = 67), and Real-Time qPCR results showed that the mRNA levels of gp96 were significantly increased in BC tissues comparing to the corresponding normal tissues (Fold changes: 2.12, **Figure 1A**). Also, the correlation of gp96 expressions with patients’ clinical features were analyzed, and our findings suggested that BC patients with higher clinical stage (III-IV), positive lymph node metastasis and higher tumor size (≥ 5cm) showed significant high-expressed gp96, but its expression status had nothing to do with patients’ age (**Table 1**). Interestingly, the BC patients were divided into two groups with (N = 34, considered as PR-BC patients) or without (N = 33, considered as PS-BC patients) paclitaxel treatment history, and we surprisingly found that gp96 mRNA levels were apparently increased in the cancer tissues collected from BC patients with paclitaxel-resistant properties in contrast with the PS-BC patients (Fold changes: 1.92, **Figure 1B**). In addition, the plasma exosomes were isolated and purified from the above BC patients’ blood samples, the morphology of the exosomes was photographed by performing the TEM assay (**Figure S1A**), and further Western Blot analysis verified that the exosome markers, including CD63 and TSG101, were expressed, but the cellular indicator β-actin was absent in the isolated exosomes (**Figure S1B**), implying that the quality of the exosomes were guaranteed. Data in **Figures 1C, D** showed that gp96 protein was upregulated in the exosomes isolated from PR-BC patients comparing to the PS-BC patients (Fold changes: 4.83). For the cellular experiments, the parental PS-BC cells (MDA-MB-453 and MCF7) were subjected to continuous low-dose paclitaxel exposure to induce the descent PR-BC cells (MDA-MB-453/PTX and MCF7/PTX) as previously reported, and the experimental procedures were shown in **Figure 1E**. The MTT assay results indicated that PR-BC cells were much more resistant to paclitaxel treatment, in contrast with the PS-BC cells (**Figures 1F–I**). Of note, comparing to the PS-BC cells, we found that low-dose paclitaxel increased gp96 expression levels in PR-BC cells at both mRNA and protein levels, as it indicated by Real-Time qPCR (**Figure 1J**) and Western Blot analysis (**Figures 1K, L**) results. Consistently, the exosomes were isolated from BC cells’ supernatants (**Figures S1A, B**), and our data supported that gp96 protein tended to be enriched in the PR-BC-exo but not in the PS-BC-exo (Fold changes: 2.83, **Figures 1M, N**). The above data suggested that intracellular and extracellular gp96 was closely associated with BC malignancy and drug-resistance.

### HSP gp96 Regulated Paclitaxel-Resistance in BC Cells in a p53-Dependent Manner

**Figure 2:**
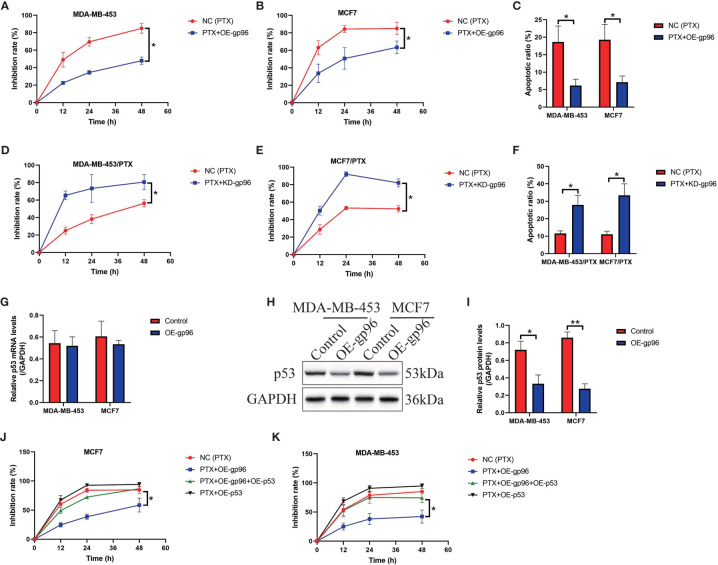
Paclitaxel-resistance was enhanced by gp96 in BC cells through degrading p53. **(A, B)** Upregulation of gp96 increased paclitaxel-resistance in PS-BC cells, as determined by MTT assay. **(C)** Overexpressed gp96 restrained paclitaxel-induced cell apoptosis in PS-BC cells (related with **Figures S3A, B**). **(D, E)** MTT assay results evidenced that silencing gp96 increased paclitaxel-sensitivity in PR-BC cells. **(F)** FCM results supported that knockdown of gp96 enhanced the promoting effects of paclitaxel on cell apoptosis in PR-BC cells (related with **Figures S3C, D**). The **(G)** mRNA and **(H, I)** protein levels of p53 in PS-BC cells were respectively examined by Real-Time qPCR and Western Blot analysis. **(J, K)** The inhibition rate of paclitaxel treatment on PS-BC cells were examined by MTT assay. Note: OE-gp69 indicated gp96 overexpression. *P < 0.05, **P < 0.01.

The following experiments were conducted to determine the role of gp96 in regulating paclitaxel-resistance in BC cells, and to achieve this, we transfected the gp96 overexpression vectors into PS-BC cells (**Figure S2A**), and the gp96 downregulation vectors were delivered into the PR-BC cells (**Figure S2B**), respectively. The vector transfection efficiency was determined by Real-Time qPCR analysis (**Figures S2A, B**). Data in **Figures 2A–C** showed that upregulation of gp96 rescued cell viability and decreased the ratio of Annexin V-FITC or PI-positive apoptotic cells in the PS-BC cells subjecting to high-dose paclitaxel stimulation, as determined by MTT assay (**Figures 2A, B**) and FCM assay (**Figure 2C** and **Figures S3A, B**), implying that gp96 overexpression increased paclitaxel-resistance in PS-BC cells. Conversely, we evidenced that knockdown of gp96 suppressed cell viability (**Figures 2D, E**) and promoted cell apoptosis (**Figure 2F**, **Figures S3C, D**) in paclitaxel-treated PR-BC cells, hinting that silence of gp96 increased paclitaxel-sensitivity in PR-BC cells. The potential underlying mechanisms were also preliminarily uncovered, and previous literatures report that p53 is the hub gene for regulating paclitaxel chemo-resistance (45, 46), and p53 can be targeted and degraded by gp96 (47), which encouraged us to assume that gp96 might regulate BC cell death through modulating p53. To investigate the effects of gp96 on p53, we conducted Real-Time qPCR and Western Blot analysis, and our results hinted that gp96 merely suppressed p53 at protein levels (Fold changes: 0.63, **Figures 2H, I**) but not in the mRNA levels (Fold changes: 1.03, **Figure 2G**) in PS-BC cells. Thus, p53 was overexpressed in PS-BC cells (**Figures S2C–E**), and the functional experiments evidenced that upregulated p53 enhanced the inhibiting effects of paclitaxel on cell viability in the PS-BC cells (**Figure 2J, K**). Also, p53 overexpression abrogated the regulating effects of gp96 overexpression on paclitaxel-resistance in PS-BC cells (**Figures 2J, K**), suggesting that intracellular gp96 controlled paclitaxel-resistance in BC cells in a p53-dependent manner.

### PR-BC Cells Derived Exosomes Delivered gp96 to Increase Paclitaxel-Resistance in PS-BC Cells

**Figure 3:**
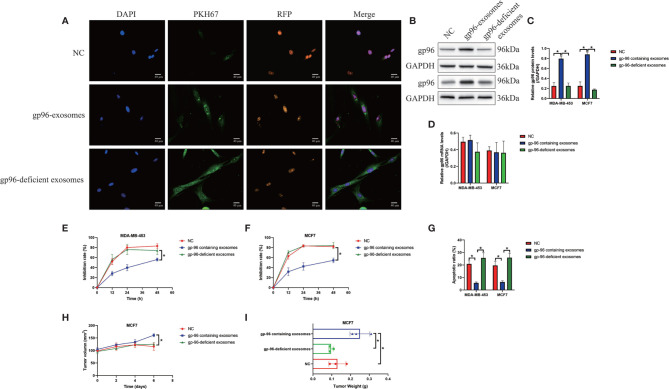
PR-BC cells derived gp96-containing exosomes increased paclitaxel-resistance in PS-BC cells. **(A)** The immunofluorescent staining assay for PKH67 and RFP indicated that the exosomes were successfully incorporated into the PS-BC cells. **(B, C)** Western Blot analysis and **(D)** Real-Time qPCR were respectively performed to examine the expression levels of gp96 at both translational and transcriptional levels. **(E, F)** Cell viability was determined by MTT assay. **(G)** Cell apoptosis was measured by FCM assay (related with **Figures S4A, B**). The xenograft tumor-bearing mice models were establishment, and **(H)** tumor volumes and **(I)** weight were monitored (related with **Figures S4C, D**). *P < 0.05.

Cancer cells derived exosomes contribute to chemo-resistance in multiple cancers, including BC (6, 7), and we next investigated whether PR-BC-exo affected paclitaxel-resistance in PS-BC cells. To explore this issue, the exosomes secreted by PR-BC cells with or without gp96-deficiency were incubated with PS-BC cells, and our data showed that PKH67-labelled exosomes translocated into the PS-BC cells (**Figure 3A**). The following Western Blot analysis evidenced that gp96 protein was successfully delivered into the PS-BC cells by PR-BC-exo (Fold changes: 3.62 and 4.32, **Figures 3B, C**), and as expected, Real-Time qPCR analysis evidenced that PR-BC-exo did not affect gp96 mRNA levels in the PS-BC cells (**Figure 3D**). Interestingly, our MTT assay data showed that PR-BC-exo with gp96 expressions was capable of increasing cell viability in paclitaxel-treated PS-BC cells (**Figures 3E, F**). Consistent with this, the FCM assay results supported that paclitaxel-induced apoptotic cell death in PS-BC cells were also reversed by co-incubating cells with PR-BC-exo (**Figure 3G** and **Figures S4A, B**). However, the PR-BC-exo with gp96-deficiency did not influence paclitaxel-resistance in PS-BC cells (**Figures 3E–G** and **Figures S4A, B**), suggesting that PR-BC-exo transmitted gp96 to increase paclitaxel-resistance in PS-BC cells. The *in vivo* experiments supported that PR-BC-exo also reversed the suppressing effects of paclitaxel on the tumorigenesis of MCF7 cells in tumor-bearing mice models (**Figures 3H, I** and **Figures S4C, D**). Those data suggested that PR-BC cells increased paclitaxel-resistance of its surrounding PS-BC cells *via* delivering gp96-containing exosomes.

### PR-BC Cells Secreted Exosomal gp96 to Activate NLRP3-Mediated Pyroptotic Cell Death in pBMSCs-Derived CD8^+^ T Cells

**Figure 4:**
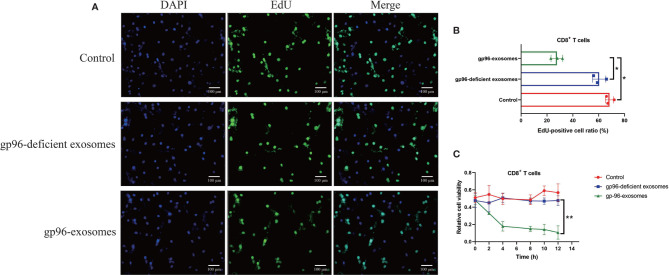
PR-BC cells derived gp96 exosomes suppressed cell viability in the CD8^+^ T cells. **(A, B)** The EdU assay was performed to examine cell proliferation abilities in the CD8^+^ T cells. **(C)** Cell viability of the CD8^+^ T cells was determined by MTT assay. *P < 0.05; **P < 0.01.

**Figure 5:**
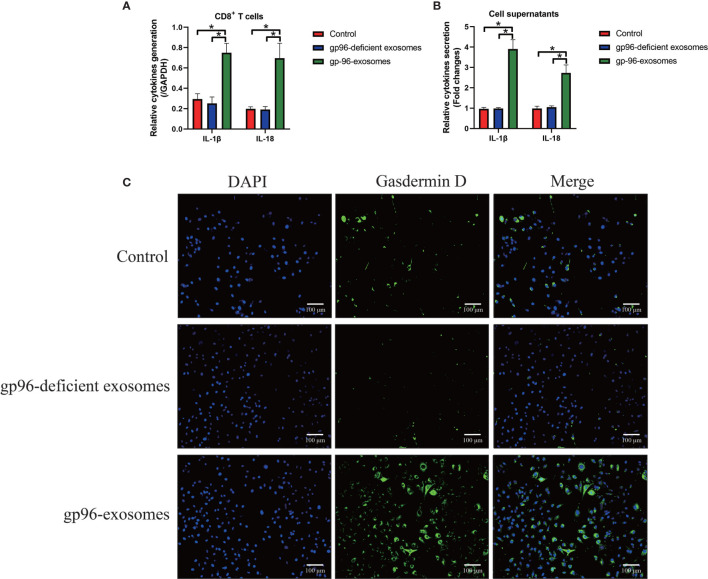
The exosomal gp96 triggered cell pyroptosis in CD8^+^ T cells. **(A)** Real-Time qPCR was performed to determine the generation of IL-1β and IL-18 in the CD8^+^ T cells. **(B)** The secretion of IL-1β and IL-18 in the CD8^+^ T cells’ supernatants were measured by ELISA. **(C)** The expression levels and cellular localization of Gasdermin D in the CD8^+^ T cells were determined by using the immunofluorescence staining assay. *P < 0.05.

As previously reported, gp96 participates in the regulation of immune evasion and surveillance during cancer progression (9), and CD8^+^ T is considered as the main immune cells to eliminate malignant cells by recognizing tumor-associated antigens in tumor cells (35, 36). However, malignant cancer cells were able to shed or hide their antigens, and a subgroup of cancer cells communicate with CD8^+^ T cells by secreting information molecules, resulting in the death of those immune cells and facilitate immune evasion (35, 36). Thus, we considered exosomal gp96 as the communicating molecule which might mediate CD8^+^ T cell death. Hence, the pBMSCs-derived CD8^+^ T cells were isolated and were co-cultured with PR-BC-exo with or without gp96 deficiency. The EdU assay (**Figures 4A, B**) and MTT assay (**Figure 4C**) were performed, and the results showed that PR-BC-exo with gp96 expression significantly suppressed cell viability and proliferation in the CD8+ T cells, while PR-BC-exo with gp96 deficiency had little effects on the above cellular functions (**Figures 4A–C**). In addition, we noticed that PR-BC-exo induced upregulation of cellular cytokines, including IL-1β and IL-18, in both CD8^+^ T cells (Fold changes: 3.45 and 4.32, **Figure 5A**) and its supernatants (Fold changes: 5.22 and 4.71, **Figure 5B**), as determined by Real-Time qPCR and ELISA. Since IL-1β and IL-18 are classical biomarkers for pyroptotic cell death, and gp96 has been proved to activate NLRP3 inflammasome in murine APCs (48), we then performed the following experiments to investigate whether exosomal gp96 triggered pyroptotic cell death in CD8^+^ T cells. As shown in **Figure 5C**, the immunofluorescent staining assay results suggested that PR-BC-exo promoted Gasdermin D expressions in CD8^+^ T cells through delivering gp96. The above results suggested that PR-BC-exo delivered gp96 to restrain cell viability in CD8^+^ T cells, which was associated with cell pyroptosis.

### Hypoxia Promoted Paclitaxel-Resistance and EMT in PS-BC Cells

**Figure 6:**
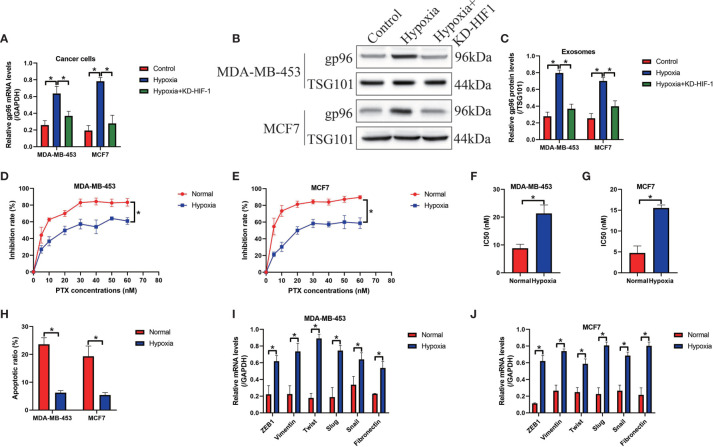
Hypoxic conditions promoted exosomal gp96 generation and increased paclitaxel-resistance in the PS-BC cells. **(A)** Hypoxia increased the mRNA levels of gp96 in PS-BC cells, as determined by Real-Time qPCR analysis. **(B, C)** Western Blot analysis was employed to detect gp96 protein levels in the PS-BC cells’ exosomes. **(D, E)** The cytotoxic effects of paclitaxel treatment on cell viability in the normal and hypoxic PS-BC cells were examined by MTT assay. **(F, G)** The IC50 values of paclitaxel in PS-BC cells were shown. **(H)** Hypoxia was capable of reversing paclitaxel-induced cell apoptosis in PS-BC cells (related with **Figures S5A, B**). **(I, J)** Hypoxic conditions promoted the expressions of the EMT associated markers in the PS-BC cells. Note: KD-HIF-1 suggested “knockdown of HIF-1”. *P < 0.05.

Hypoxia is common phenomena in solid tumors when tumor volume reaches about 2 mm, and hypoxic pressure brings significant changes to the cellular functions of both tumor and stromal cells in the tumor microenvironment, which further facilitates angiogenesis and cancer metastasis (13–15). Of note, recent data indicate that hypoxia increases gp96 expressions through upregulating HIF-1 (9), which were verified in our study. Specifically, the PS-BC cells were subjected to HIF-1 knockdown vectors (**Figure S6**) and hypoxic treatments, and the cancer cells and their exosomes in the supernatants were collected. By performing Real-Time qPCR (**Figure 6A**) and Western Blot analysis (**Figures 6B, C**), we verified that hypoxia promoted gp96 generation (Fold changes: 3.22 and 4.01) and secretion (Fold changes: 2.34 and 2.91) in the PS-BC cells and supernatants, which were reversed by silencing HIF-1, suggesting that hypoxia promoted intracellular and extracellular gp96 expressions through inducing HIF-1 expressions. Then, we explored the paclitaxel-resistant characteristics of the PS-BC cells under normal or hypoxic environment (**Figures 6D–H** and **Figures S5A, B**). To achieve this, the mentioned PS-BC cells were subjected to high-dose paclitaxel treatment, and the MTT assay supported that paclitaxel especially suppressed cell viability in PS-BC cells without hypoxic stimulation (**Figures 6D, E**). Also, the FCM results evidenced that hypoxic PS-BC cells were much more resistant to paclitaxel-induced cell apoptosis than the normal PS-BC cells (**Figures 6F, G** and **Figures S5A, B**), suggesting that hypoxia increased paclitaxel-resistant properties in PS-BC cells. Moreover, we profiled the EMT associated makers in the PS-BC cells, and the Real-Time qPCR analysis results validated that the mRNA levels of the mesenchymal biomarkers, including ZEB1, vimentin, twist, slug, snail and fibronectin, were all elevated by hypoxic stimulations in PS-BC cells (**Figures 6I, J**). Taken together those data, we concluded that Hypoxia affected gp96 expressions and secretion in BC cells *via* inducing HIF-1, and hypoxic condition was capable of increasing paclitaxel-resistance and promoting EMT process in the PS-BC cells.

## Discussion

Intracellular and extracellular gp96 is closely associated with cancer aggressiveness (10, 11) and drug resistance (12), but the molecular mechanisms by which gp96 controls cancer progression and paclitaxel-resistance in BC have not been investigated. According to the previous publications, gp96 serves as an oncogene to facilitate BC progression (10, 11) and enhance paclitaxel-resistance in ovarian cancer (12), which are supported by our results that gp96 was upregulated in the paclitaxel-resistant BC (PR-BC) tissues and cells, in contrast with their paclitaxel-sensitive BC (PS-BC) counterparts. To validate the biological functions of gp96, we conducted the following functional experiments and evidenced that overexpression of gp96 increased paclitaxel-resistance in PS-BC cells, whereas gp96 ablation sensitized PR-BC cells to paclitaxel treatments, suggesting that gp96 promoted paclitaxel-resistance in BC, which were supported by the previous publications (10–12). Moreover, we verified that gp96 regulated paclitaxel-resistance in BC cells through modulating p53. As previously described, gp96 decreased p53 stability to promote its degradation in liver cancer (47), and inhibition of gp96 by its inhibitor upregulated p53 expressions to suppress Hepatitis B (HBV) replication (49). In addition, depletion of p53 increases paclitaxel-resistance in BC (46, 50, 51), indicating that existence of p53 sustains paclitaxel-sensitivity in BC. Based on the above information, we further evidenced that gp96 degraded p53 proteins instead of its mRNA in BC, and the promoting effects of gp96 overexpression on paclitaxel-resistance were abrogated by upregulating p53, which were in consistent with the previous publications (46, 50, 51).

In addition to the intracellular gp96, recent studies suggest that gp96 is incorporated into the exosomes to act as informative molecules for cell-to-cell communication, which is pivotal for sustaining chemoresistance in multiple cancers (8, 9). In our experiments, we isolated the BC cells derived exosomes, and found that gp96 was significantly enriched in the PR-BC cell derived exosomes, instead of the PS-BC cells derived exosomes. Furthermore, through conducting *in vitro* and *in vivo* experiments, we found that gp96-containing exosomes increased paclitaxel-resistance in PS-BC cells, hinting that PR-BC cells delivered exosomal gp96 to increase paclitaxel-resistance in PS-BC cells. In addition, previous literatures report that cancer cells derived exosomes interact with immune cells to promote immune evasion during cancer pathogenesis (37–39), and researchers notice that gp96 is considered as biomarker for immune surveillance *via* influencing CD4^+^ and CD8^+^ T-lymphocytes (9, 40), which are supported by our results that gp96-containing exosomes suppressed cell viability in the CD8^+^ T cells, which is the main immune cells that recognize tumor antigens (35, 36). Also, we verified that extracellular gp96 triggered pyroptotic cell death in CD8^+^ T cells, which is indirectly validated by the available information that gp96 activated NLRP3 inflammasome in the antigen presenting cells (APCs) (48).

Hypoxia is common phenomena in solid tumors, and deprivation of oxygen and nutrients under this situation make a small subgroup of cancer cells in the inner tumors more aggressiveness (16–18) and even resistant to chemical drugs (19, 52–55). For example, Liu et al. evidence that hypoxia facilitates chemoresistance in glioma (19), Xu et al. report that hypoxia induces chemoresistance in non-small cell lung cancer (NSCLC) (55), and Zhu et al. find that cisplatin-resistance in esophageal cancer cells was also enhanced by hypoxic conditions (54). Of note, hypoxia also promotes paclitaxel-resistance in multiple cancers (52, 53), which are supported by our results that hypoxia promoted paclitaxel-resistance in PS-BC cells. In addition, hypoxia is related with cancer metastasis (16–18), and we evidenced that hypoxia accelerated EMT process in PS-BC cells. Moreover, as previously reported, hypoxia influences cell-to-cell communications in tumor microenvironment *via* promoting exosomes secretion (56), and hypoxia induces gp96 upregulation (26). Based on this, we reported that hypoxia promoted exosomal gp96 generation in PS-BC cells through upregulating HIF-1, which are evidenced by the available information that HIF-1 is capable of elevating gp96 (9, 31).

## Conclusions

Thus, we summarized the findings of this study as follows: (1) Both continuous long-term paclitaxel exposure and hypoxic conditions induced gp96 upregulation and secretion. (2) The intracellular gp96 degraded p53 and resulted in paclitaxel-resistance in PS-BC cells. (3) The extracellular gp96-containing exosomes transformed the PS-BC cells into PR-BC cells. (4) PR-BC cells derived gp96-containing exosomes initiated pyroptotic cell death in CD8^+^ T cells to facilitate immune evasion.

## Data Availability Statement

The original contributions presented in the study are included in the article/**Supplementary Material**. Further inquiries can be directed to the corresponding author.

## Ethics Statement

The studies involving human participants were reviewed and approved by Ethics Committee Affiliated to Harbin Medical University Cancer Hospital. The patients/participants provided their written informed consent to participate in this study. The animal study was reviewed and approved by Ethics Committee Affiliated to Harbin Medical University Cancer Hospital.

## Author Contributions

TT and JGH were co-first authors, and they contributed equally to this work. They were responsible for the conception, investigations, experiments, software, data collection/analysis, and they also drafted the initial version of the manuscript. JH and SL contributed to this work in terms of data analysis and visualization, they also revised the manuscript regarding to the grammatical problems. HP was the corresponding author of this study, and was responsible for the conception, guidance, funding acquisition, manuscript proofreading and submission. All authors contributed to the article and approved the submitted version.

## Funding

This study was financially supported by the fundings as follows:

(1) Natural Science and Technology Fund of Heilongjiang Province of China (Grant Number LH2020H126).

(2) Hai Yan Fund from Harbin Medical University Cancer Hospital (Grant Number JJZD2020-06).

(3) National Cancer Center climbing Foundation (Grant Number NCC201808B014).

(4) Wu Jieping Medical Foundation (Grant Number 320.6750.18215).

(5) Heilongjiang Postdoctoral Fund (Grant Number LBH- Q18096).

## Conflict of Interest

The authors declare that the research was conducted in the absence of any commercial or financial relationships that could be construed as a potential conflict of interest.

## Publisher’s Note

All claims expressed in this article are solely those of the authors and do not necessarily represent those of their affiliated organizations, or those of the publisher, the editors and the reviewers. Any product that may be evaluated in this article, or claim that may be made by its manufacturer, is not guaranteed or endorsed by the publisher.

## Supplementary Material

Supplementary Figure 1 Isolation, purification and identification of the exosomes. **(A)** TEM was conducted to determine the morphologies of the isolated exosomes. **(B)** Western Blot analysis was used to examine the expression levels of the exosome-associated biomarkers.

Supplementary Figure 2 The vector transfection efficiency of **(A)** gp96 overexpression in PS-BC cells, **(B)** gp96 downregulation in PR-BC cells, and **(C–E)** p53 ablation in PS-BC cells, were measured by performing Real-Time qPCR and Western Blot analysis. ***P* < 0.01; ****P* < 0.001.

Supplementary Figure 3 FCM was used to examine cell apoptosis ratio in the **(A, B)** PS-BC cells and **(C, D)** PR-BC cells, respectively. **P* < 0.05.

Supplementary Figure 4
**(A, B)** FCM was performed to determine cell apoptosis in PS-BC cells. **(C, D)** Tumor volumes of the xenograft tumor-bearing mice models were photographed and measured. **P* < 0.05.

Supplementary Figure 5 Cell apoptosis ratio in the PS-BC cells were determined by FCM analysis. **P* < 0.05.

Supplementary Figure 6 The transfection efficiency of HIF-1 knockdown vectors was determined by performing Real-Time qPCR analysis. **P* < 0.05

## References

[1] Liu G Zhang Z Song Q Guo Y Bao P Shui H. Circ_0006528 Contributes to Paclitaxel Resistance of Breast Cancer Cells by Regulating miR-1299/CDK8 Axis. Onco Targets Ther (2020) 13:9497–511. doi: 10.2147/OTT.S252886 PMC7522311 33061434

[2] Yang W Gong P Yang Y Yang C Yang B Ren L. Circ-ABCB10 Contributes to Paclitaxel Resistance in Breast Cancer Through Let-7a-5p/DUSP7 Axis. Cancer Manag Res (2020) 12:2327–37. doi: 10.2147/CMAR.S238513 PMC7108723 32273769

[3] Diéras V Han HS Kaufman B Wildiers H Friedlander M Ayoub JP. Veliparib With Carboplatin and Paclitaxel in BRCA-Mutated Advanced Breast Cancer (BROCADE3): A Randomised, Double-Blind, Placebo-Controlled, Phase 3 Trial. Lancet Oncol (2020) 21(10):1269–82. doi: 10.1016/S1470-2045(20)30447-2 32861273

[4] Schmid P Rugo HS Adams S Schneeweiss A Barrios CH Iwata H. Atezolizumab Plus Nab-Paclitaxel as First-Line Treatment for Unresectable, Locally Advanced or Metastatic Triple-Negative Breast Cancer (IMpassion130): Updated Efficacy Results From a Randomised, Double-Blind, Placebo-Controlled, Phase 3 Trial. Lancet Oncol (2020) 21(1):44–59. doi: 10.1016/S1470-2045(19)30689-8 31786121

[5] Tolaney SM Guo H Pernas S Barry WT Dillon DA Ritterhouse L. Seven-Year Follow-Up Analysis of Adjuvant Paclitaxel and Trastuzumab Trial for Node-Negative, Human Epidermal Growth Factor Receptor 2-Positive Breast Cancer. J Clin Oncol (2019) 37(22):1868–75. doi: 10.1200/JCO.19.00066 PMC7587424 30939096

[6] Pederson PJ Liang H Filonov D Mooberry SL. Eribulin and Paclitaxel Differentially Alter Extracellular Vesicles and Their Cargo From Triple-Negative Breast Cancer Cells. Cancers (Basel) (2021) 13(11):2783. doi: 10.3390/cancers13112783 34205051 PMC8199867

[7] Yang Q Zhao S Shi Z Cao L Liu J Pan T. Chemotherapy-Elicited Exosomal miR-378a-3p and miR-378d Promote Breast Cancer Stemness and Chemoresistance *via* the Activation of EZH2/STAT3 Signaling. J Exp Clin Cancer Res (2021) 40(1):120. doi: 10.1186/s13046-021-01901-1 33823894 PMC8022546

[8] Cianciaruso C Phelps EA Pasquier M Hamelin R Demurtas D Alibashe Ahmed M. Primary Human and Rat β-Cells Release the Intracellular Autoantigens GAD65, IA-2, and Proinsulin in Exosomes Together With Cytokine-Induced Enhancers of Immunity. Diabetes (2017) 66(2):460–73. doi: 10.2337/db16-0671 27872147

[9] Taha EA Ono K Eguchi T. Roles of Extracellular HSPs as Biomarkers in Immune Surveillance and Immune Evasion. Int J Mol Sci (2019) 20(18):4588. doi: 10.3390/ijms20184588 PMC6770223 31533245

[10] Li X Sun L Hou J Gui M Ying J Zhao H. Cell Membrane Gp96 Facilitates HER2 Dimerization and Serves as a Novel Target in Breast Cancer. Int J Cancer (2015) 137(3):512–24. doi: 10.1002/ijc.29405 25546612

[11] Li X Wang B Liu W Gui M Peng Z Meng S. Blockage of Conformational Changes of Heat Shock Protein Gp96 on Cell Membrane by a α-Helix Peptide Inhibits HER2 Dimerization and Signaling in Breast Cancer. PloS One (2015) 10(4):e0124647. doi: 10.1371/journal.pone.0124647 25898135 PMC4405268

[12] Di Michele M Marcone S Cicchillitti L Della Corte A Ferlini C Scambia G. Glycoproteomics of Paclitaxel Resistance in Human Epithelial Ovarian Cancer Cell Lines: Towards the Identification of Putative Biomarkers. J Proteomics (2010) 73(5):879–98. doi: 10.1016/j.jprot.2009.11.012 19951750

[13] Chen XJ Deng YR Wang ZC Wei WF Zhou CF Zhang YM. Hypoxia-Induced ZEB1 Promotes Cervical Cancer Progression *via* CCL8-Dependent Tumour-Associated Macrophage Recruitment. Cell Death Dis (2019) 10(7):508. doi: 10.1038/s41419-019-1748-1 31263103 PMC6602971

[14] Qureshi-Baig K Kuhn D Viry E Pozdeev VI Schmitz M Rodriguez F. Hypoxia-Induced Autophagy Drives Colorectal Cancer Initiation and Progression by Activating the PRKC/PKC-EZR (Ezrin) Pathway. Autophagy (2020) 16(8):1436–52. doi: 10.1080/15548627.2019.1687213 PMC7469473 31775562

[15] Vaupel P Schmidberger H Mayer A. The Warburg Effect: Essential Part of Metabolic Reprogramming and Central Contributor to Cancer Progression. Int J Radiat Biol (2019) 95(7):912–9. doi: 10.1080/09553002.2019.1589653 30822194

[16] Niu Y Bao L Chen Y Wang C Luo M Zhang B. HIF2-Induced Long Noncoding RNA RAB11B-AS1 Promotes Hypoxia-Mediated Angiogenesis and Breast Cancer Metastasis. Cancer Res (2020) 80(5):964–75. doi: 10.1158/0008-5472.CAN-19-1532 PMC7056556 31900259

[17] Schito L. Hypoxia-Dependent Angiogenesis and Lymphangiogenesis in Cancer. Adv Exp Med Biol (2019) 1136:71–85. doi: 10.1007/978-3-030-12734-3_5 31201717

[18] Semenza GL. Cancer-Stromal Cell Interactions Mediated by Hypoxia-Inducible Factors Promote Angiogenesis, Lymphangiogenesis, and Metastasis. Oncogene (2013) 32(35):4057–63. doi: 10.1038/onc.2012.578 PMC4415159 23222717

[19] Liu J Gao L Zhan N Xu P Yang J Yuan F. Hypoxia Induced Ferritin Light Chain (FTL) Promoted Epithelia Mesenchymal Transition and Chemoresistance of Glioma. J Exp Clin Cancer Res (2020) 39(1):137. doi: 10.1186/s13046-020-01641-8 32677981 PMC7364815

[20] Ling S Shan Q Zhan Q Ye Q Liu P Xu S. USP22 Promotes Hypoxia-Induced Hepatocellular Carcinoma Stemness by a HIF1α/USP22 Positive Feedback Loop Upon TP53 Inactivation. Gut (2020) 69(7):1322–34. doi: 10.1136/gutjnl-2019-319616 31776228

[21] Wu H Chu Y Sun S Li G Xu S Zhang X. Hypoxia-Mediated Complement 1q Binding Protein Regulates Metastasis and Chemoresistance in Triple-Negative Breast Cancer and Modulates the PKC-NF-κb-VCAM-1 Signaling Pathway. Front Cell Dev Biol (2021) 9:607142. doi: 10.3389/fcell.2021.607142 33708767 PMC7940382

[22] Tang K Zhu L Chen J Wang D Zeng L Chen C. Hypoxia Promotes Breast Cancer Cell Growth by Activating a Glycogen Metabolic Program. Cancer Res (2021) 81(19):4949–63. doi: 10.1158/0008-5472.CAN-21-0753 34348966

[23] Maciel L de Oliveira DF Monnerat G Campos de Carvalho AC Nascimento JHM. Exogenous 10 kDa-Heat Shock Protein Preserves Mitochondrial Function After Hypoxia/Reoxygenation. Front Pharmacol (2020) 11:545. doi: 10.3389/fphar.2020.00545 32431608 PMC7214810

[24] Madaeva IM Kurashova NA Semenova NV Ukhinov EB Kolesnikov SI Kolesnikova LI. Heat Shock Protein HSP70 in Oxidative Stress in Apnea Patients. Bull Exp Biol Med (2020) 169(5):695–7. doi: 10.1007/s10517-020-04957-9 32986213

[25] Zhang G Cheng W Du L Xu C Li J. Synergy of Hypoxia Relief and Heat Shock Protein Inhibition for Phototherapy Enhancement. J Nanobiotech (2021) 19(1):9. doi: 10.1186/s12951-020-00749-5 PMC7789325 33407570

[26] Kutomi G Tamura Y Okuya K Yamamoto T Hirohashi Y Kamiguchi K. Targeting to Static Endosome Is Required for Efficient Cross-Presentation of Endoplasmic Reticulum-Resident Oxygen-Regulated Protein 150-Peptide Complexes. J Immunol (2009) 183(9):5861–9. doi: 10.4049/jimmunol.0803768 19812200

[27] Chen L Bao L Niu Y Wang JE Kumar A Xing C. LncIHAT Is Induced by Hypoxia-Inducible Factor 1 and Promotes Breast Cancer Progression. Mol Cancer Res (2021) 19(4):678–87. doi: 10.1158/1541-7786.MCR-20-0383 33380467

[28] Xiang L Semenza GL. Hypoxia-Inducible Factors Promote Breast Cancer Stem Cell Specification and Maintenance in Response to Hypoxia or Cytotoxic Chemotherapy. Adv Cancer Res (2019) 141:175–212. doi: 10.1016/bs.acr.2018.11.001 30691683

[29] Zhang HS Zhang ZG Du GY Sun HL Liu HY Zhou Z. Nrf2 Promotes Breast Cancer Cell Migration *via* Up-Regulation of G6PD/HIF-1α/Notch1 Axis. J Cell Mol Med (2019) 23(5):3451–63. doi: 10.1111/jcmm.14241 PMC6484400 30809937

[30] Yen TY Stephen ZR Lin G Mu Q Jeon M Untoro S. Catalase-Functionalized Iron Oxide Nanoparticles Reverse Hypoxia-Induced Chemotherapeutic Resistance. Adv Healthc Mater (2019) 8(20):e1900826. doi: 10.1002/adhm.201900826 31557421 PMC6919328

[31] Singhal S Wiewrodt R Malden LD Amin KM Matzie K Friedberg J. Gene Expression Profiling of Malignant Mesothelioma. Clin Cancer Res (2003) 9(8):3080–97. 12912960

[32] Lei X Lei Y Li JK Du WX Li RG Yang J. Immune Cells Within the Tumor Microenvironment: Biological Functions and Roles in Cancer Immunotherapy. Cancer Lett (2020) 470:126–33. doi: 10.1016/j.canlet.2019.11.009 31730903

[33] Liu H Kuang X Zhang Y Ye Y Li J Liang L. ADORA1 Inhibition Promotes Tumor Immune Evasion by Regulating the ATF3-PD-L1 Axis. Cancer Cell (2020) 37(3):324–39.e8. doi: 10.1016/j.ccell.2020.02.006 32183950

[34] Yamamoto K Venida A Yano J Biancur DE Kakiuchi M Gupta S. Autophagy Promotes Immune Evasion of Pancreatic Cancer by Degrading MHC-I. Nature (2020) 581(7806):100–5. doi: 10.1038/s41586-020-2229-5 PMC7296553 32376951

[35] Hong W Xue M Jiang J Zhang Y Gao X. Circular RNA Circ-CPA4/ Let-7 miRNA/PD-L1 Axis Regulates Cell Growth, Stemness, Drug Resistance and Immune Evasion in Non-Small Cell Lung Cancer (NSCLC). J Exp Clin Cancer Res (2020) 39(1):149. doi: 10.1186/s13046-020-01648-1 32746878 PMC7397626

[36] Liu Z Zhou Q Wang Z Zhang H Zeng H Huang Q. Intratumoral TIGIT(+) CD8(+) T-Cell Infiltration Determines Poor Prognosis and Immune Evasion in Patients With Muscle-Invasive Bladder Cancer. J Immunother Cancer (2020) 8(2):e000978. doi: 10.1136/jitc-2020-000978 32817209 PMC7430558

[37] Chen G Huang AC Zhang W Zhang G Wu M Xu W. Exosomal PD-L1 Contributes to Immunosuppression and is Associated With Anti-PD-1 Response. Nature (2018) 560(7718):382–6. doi: 10.1038/s41586-018-0392-8 PMC6095740 30089911

[38] Kulkarni B Kirave P Gondaliya P Jash K Jain A Tekade RK. Exosomal miRNA in Chemoresistance, Immune Evasion, Metastasis and Progression of Cancer. Drug Discovery Today (2019) 24(10):2058–67. doi: 10.1016/j.drudis.2019.06.010 31228614

[39] Qadir F Aziz MA Sari CP Ma H Dai H Wang X. Transcriptome Reprogramming by Cancer Exosomes: Identification of Novel Molecular Targets in Matrix and Immune Modulation. Mol Cancer (2018) 17(1):97. doi: 10.1186/s12943-018-0846-5 30008265 PMC6047127

[40] Radolovic P Grebic D Mustac E Sebaher I Mamic J Miletic WM. Heat Shock Protein Gp96 and CD4+ and CD8+ T-Lymphocytes Expression as Prognostic Factors in Various Molecular Types of Invasive Breast Carcinoma. Neoplasma (2020) 67(2):421–9. doi: 10.4149/neo_2020_190601N478 31973538

[41] You L Wu W Wang X Fang L Adam V Nepovimova E. The Role of Hypoxia-Inducible Factor 1 in Tumor Immune Evasion. Med Res Rev (2021) 41(3):1622–43. doi: 10.1002/med.21771 33305856

[42] Han J Han B Wu X Hao J Dong X Shen Q. Knockdown of lncRNA H19 Restores Chemo-Sensitivity in Paclitaxel-Resistant Triple-Negative Breast Cancer Through Triggering Apoptosis and Regulating Akt Signaling Pathway. Toxicol Appl Pharmacol (2018) 359:55–61. doi: 10.1016/j.taap.2018.09.018 30244121

[43] Zhang X Sai B Wang F Wang L Wang Y Zheng L. Hypoxic BMSC-Derived Exosomal miRNAs Promote Metastasis of Lung Cancer Cells *via* STAT3-Induced EMT. Mol Cancer (2019) 18(1):40. doi: 10.1186/s12943-019-0959-5 30866952 PMC6417285

[44] Wang X He Q Shen H Xia A Tian W Yu W. TOX Promotes the Exhaustion of Antitumor CD8(+) T Cells by Preventing PD1 Degradation in Hepatocellular Carcinoma. J Hepatol (2019) 71(4):731–41. doi: 10.1016/j.jhep.2019.05.015 31173813

[45] Parmakhtiar B Burger RA Kim JH Fruehauf JP. HIF Inactivation of P53 in Ovarian Cancer Can Be Reversed by Topotecan, Restoring Cisplatin and Paclitaxel Sensitivity. Mol Cancer Res (2019) 17(8):1675–86. doi: 10.1158/1541-7786.MCR-18-1109 31088908

[46] Xue J Chi Y Chen Y Huang S Ye X Niu J. MiRNA-621 Sensitizes Breast Cancer to Chemotherapy by Suppressing FBXO11 and Enhancing P53 Activity. Oncogene (2016) 35(4):448–58. doi: 10.1038/onc.2015.96 PMC4603999 25867061

[47] Wu B Chu X Feng C Hou J Fan H Liu N. Heat Shock Protein Gp96 Decreases P53 Stability by Regulating Mdm2 E3 Ligase Activity in Liver Cancer. Cancer Lett (2015) 359(2):325–34. doi: 10.1016/j.canlet.2015.01.034 25637791

[48] Wang Y Sedlacek AL Pawaria S Xu H Scott MJ Binder RJ. Cutting Edge: The Heat Shock Protein Gp96 Activates Inflammasome-Signaling Platforms in APCs. J Immunol (2018) 201(8):2209–14. doi: 10.4049/jimmunol.1800505 PMC6176107 30209191

[49] Qian L Fan H Ju Y Chen L Li X Ye X. A Peptide-Based Inhibitor of Gp96 Suppresses HBsAg Expression and HBV Replication by Upregulation of P53. J Gen Virol (2019) 100(8):1241–52. doi: 10.1099/jgv.0.001289 31204972

[50] Aborehab NM Elnagar MR Waly NE. Gallic Acid Potentiates the Apoptotic Effect of Paclitaxel and Carboplatin *via* Overexpression of Bax and P53 on the MCF-7 Human Breast Cancer Cell Line. J Biochem Mol Toxicol (2021) 35(2):e22638. doi: 10.1002/jbt.22638 33002289

[51] Khordadmehr M Shahbazi R Baradaran B Sadreddini S Shanebandi D Hajiasgharzadeh K. Restoring of miR-193a-5p Sensitizes Breast Cancer Cells to Paclitaxel Through P53 Pathway. Adv Pharm Bull (2020) 10(4):595–601. doi: 10.34172/apb.2020.071 33072537 PMC7539307

[52] Feng L Shen F Zhou J Li Y Jiang R Chen Y. Hypoxia-Induced Up-Regulation of miR-27a Promotes Paclitaxel Resistance in Ovarian Cancer. Biosci Rep (2020) 40(4):BSR20192457. doi: 10.1042/BSR20192457 32190895 PMC7109003

[53] Li K Li M Luo Z Mao Y Yu Y He Y. Overcoming the Hypoxia-Induced Drug Resistance in Liver Tumor by the Concurrent Use of Apigenin and Paclitaxel. Biochem Biophys Res Commun (2020) 526(2):321–7. doi: 10.1016/j.bbrc.2020.03.010 32220496

[54] Zhu ZJ Pang Y Jin G Zhang HY Wang WH Liu JW. Hypoxia Induces Chemoresistance of Esophageal Cancer Cells to Cisplatin Through Regulating the lncRNA-EMS/miR-758-3p/WTAP Axis. Aging (Albany NY) (2021) 13(13):17155–76. doi: 10.18632/aging.203062 PMC8312407 34081626

[55] Xu R Luo X Ye X Li H Liu H Du Q. SIRT1/PGC-1α/PPAR-γ Correlate With Hypoxia-Induced Chemoresistance in Non-Small Cell Lung Cancer. Front Oncol (2021) 11:682762. doi: 10.3389/fonc.2021.682762 34381712 PMC8351465

[56] Yang H Zhang H Yang Y Wang X Deng T Liu R. Hypoxia Induced Exosomal circRNA Promotes Metastasis of Colorectal Cancer *via* Targeting GEF-H1/RhoA Axis. Theranostics (2020) 10(18):8211–26. doi: 10.7150/thno.44419 PMC7381736 32724467

