# Supplementary material for: Hypoxia-Induced Intracellular and Extracellular Heat Shock Protein gp96 Increases Paclitaxel-Resistance and Facilitates Immune Evasion in Breast Cancer
Source: Front Oncol. 2021 Dec 20;11:784777. doi: 10.3389/fonc.2021.784777 (PMC8722103; doi:10.3389/fonc.2021.784777)
Supplement: Supplementary Figure 1 — Isolation, purification and identification of the exosomes. (A) TEM was conducted to determine the morphologies of the isolated exosomes. (B) Western Blot analysis was used to examine the expression levels of the exosome-associated biomarkers. [file DataSheet_1.docx]

**Supplementary Figures and Figure legends**


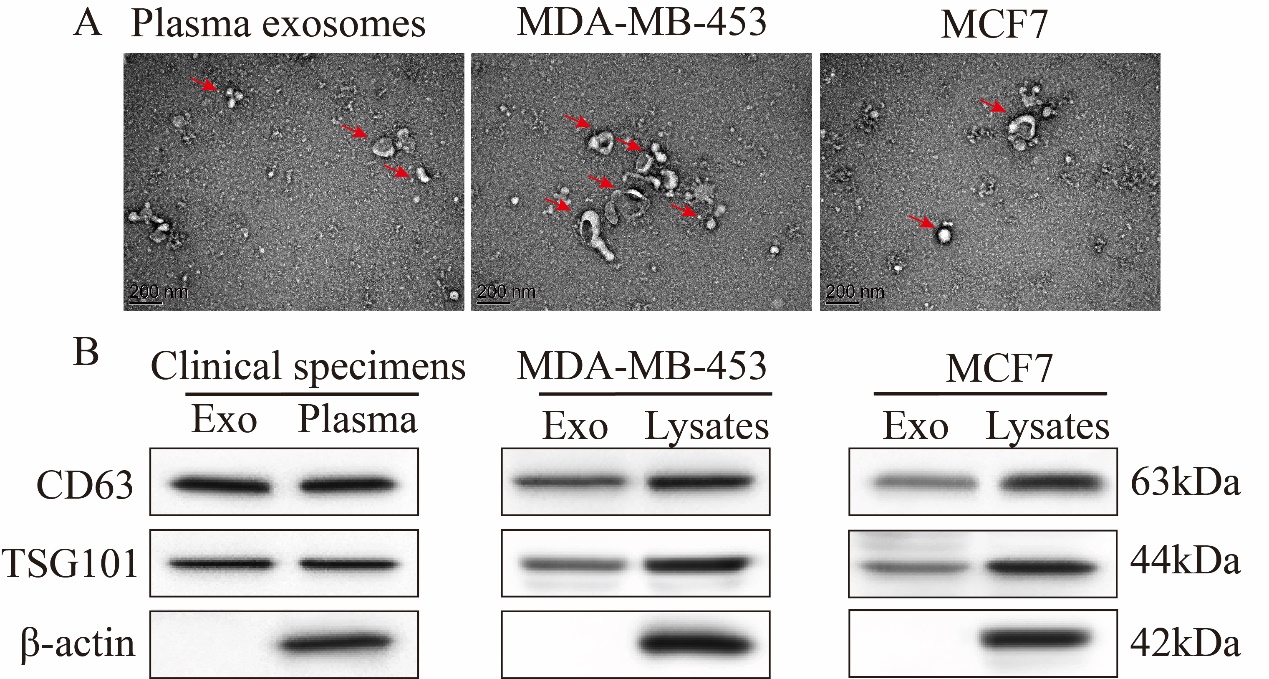


**Figure S1.** Isolation, purification and identification of the exosomes. (A) TEM was conducted to determine the morphologies of the isolated exosomes. (B) Western Blot analysis was used to examine the expression levels of the exosome-associated biomarkers.


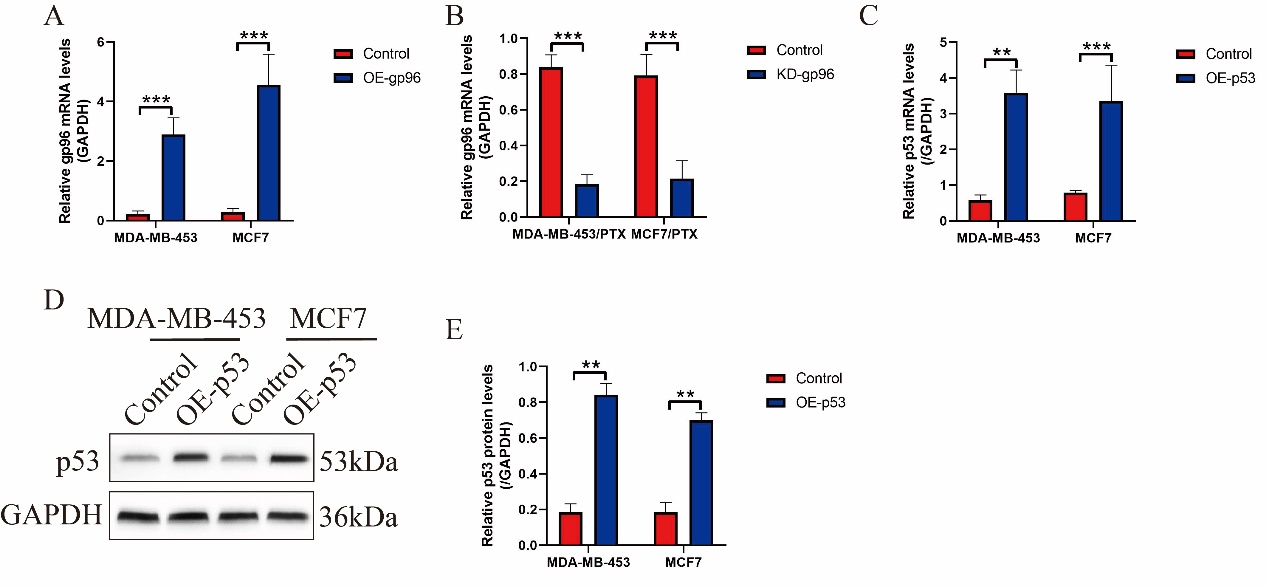


**Figure S2.** The vector transfection efficiency of (A) gp96 overexpression in PS-BC cells, (B) gp96 downregulation in PR-BC cells, and (C-E) p53 ablation in PS-BC cells, were measured by performing Real-Time qPCR and Western Blot analysis. ***P* < 0.01; ****P* < 0.001.


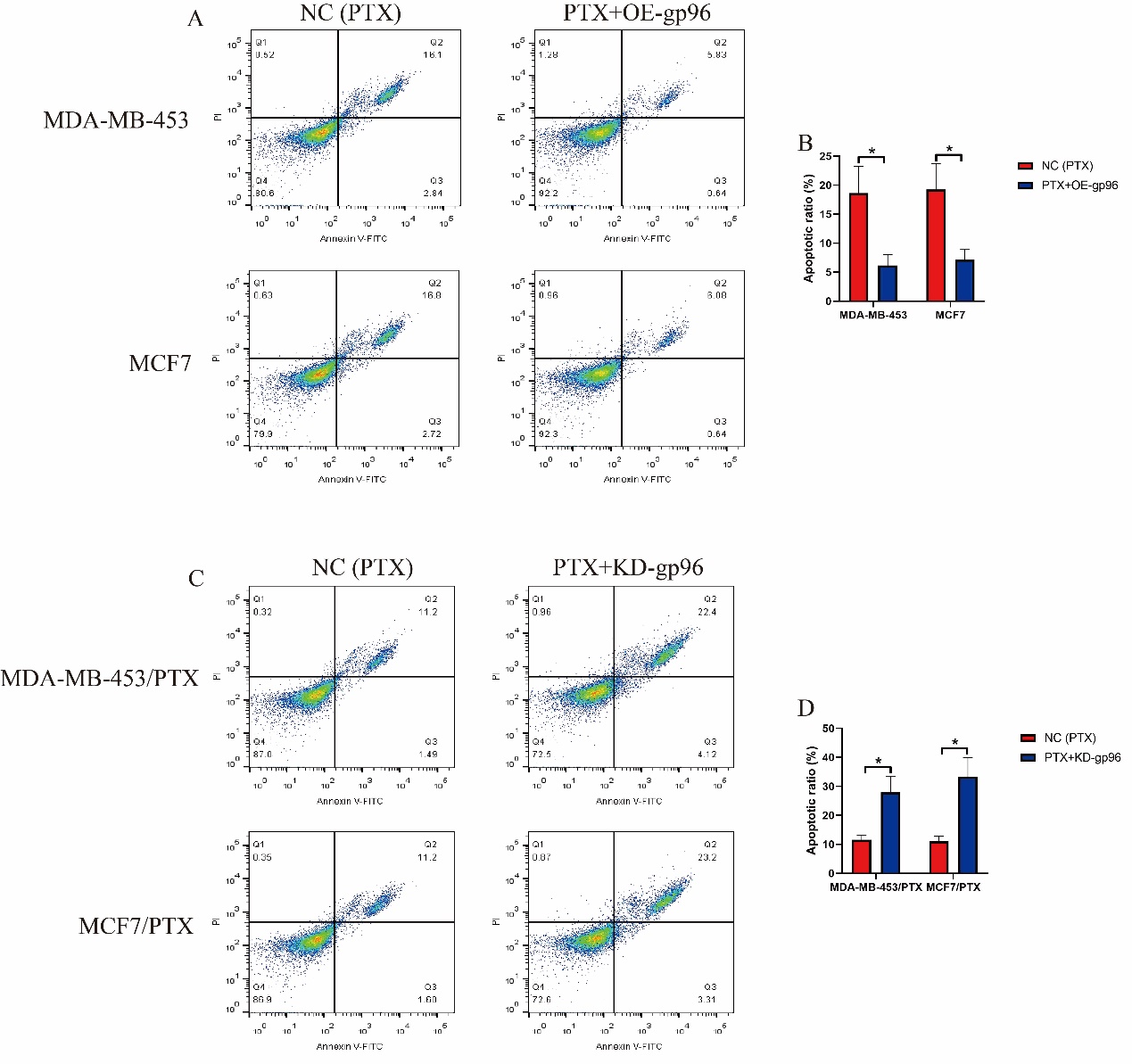


**Figure S3.** FCM was used to examine cell apoptosis ratio in the (A, B) PS-BC cells and (C, D) PR-BC cells, respectively. **P* < 0.05.


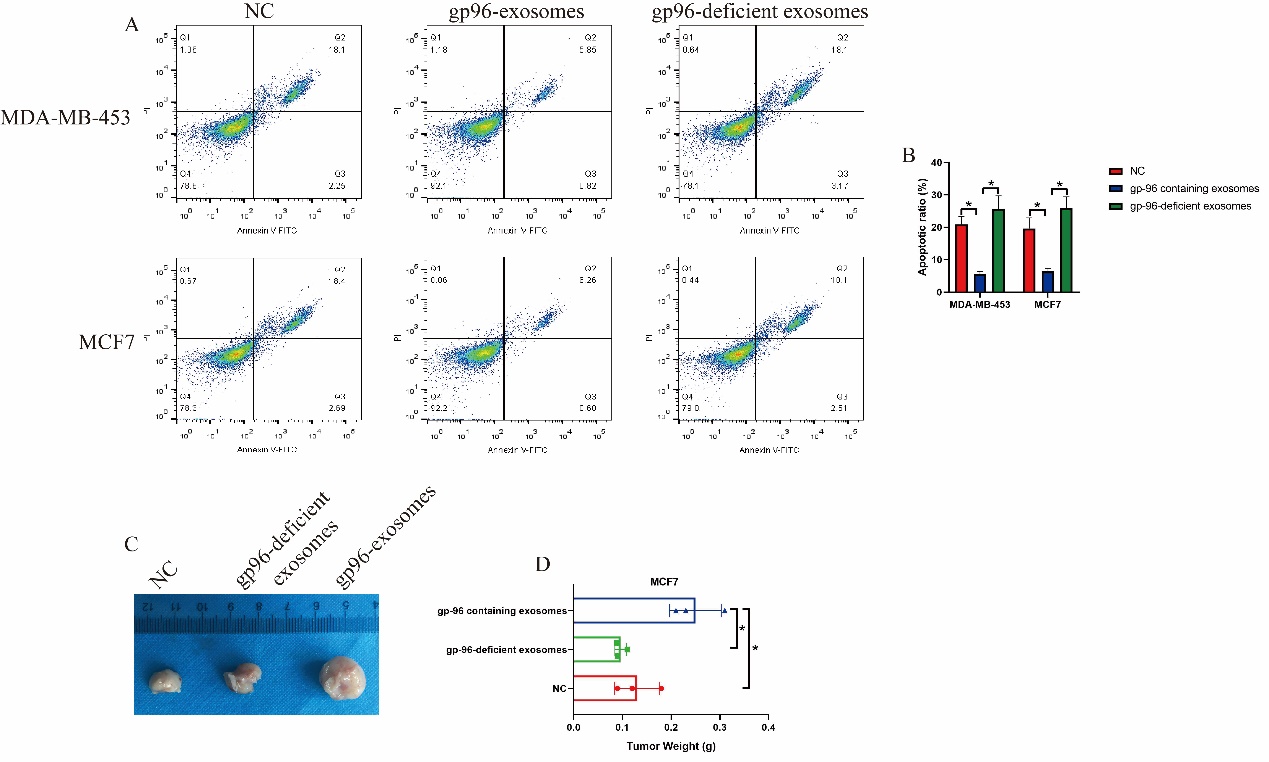


**Figure S4.** (A, B) FCM was performed to determine cell apoptosis in PS-BC cells. (C, D) Tumor volumes of the xenograft tumor-bearing mice models were photographed and measured. **P* < 0.05.


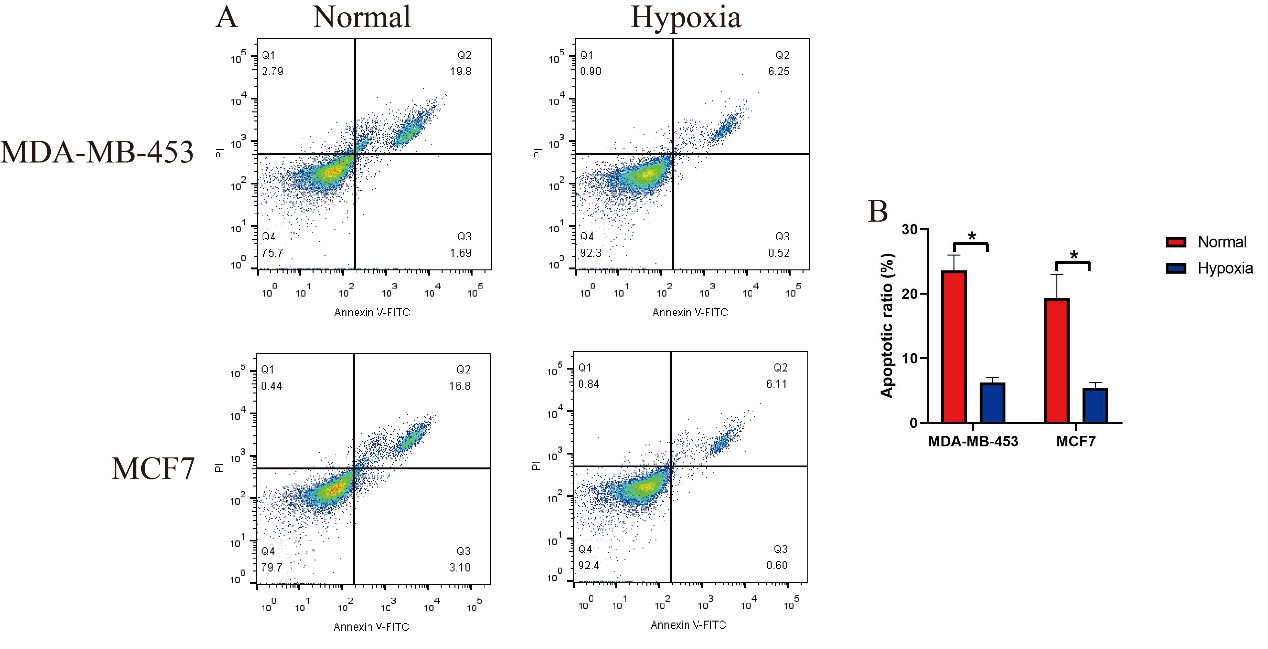


**Figure S5.** Cell apoptosis ratio in the PS-BC cells were determined by FCM analysis. **P* < 0.05.


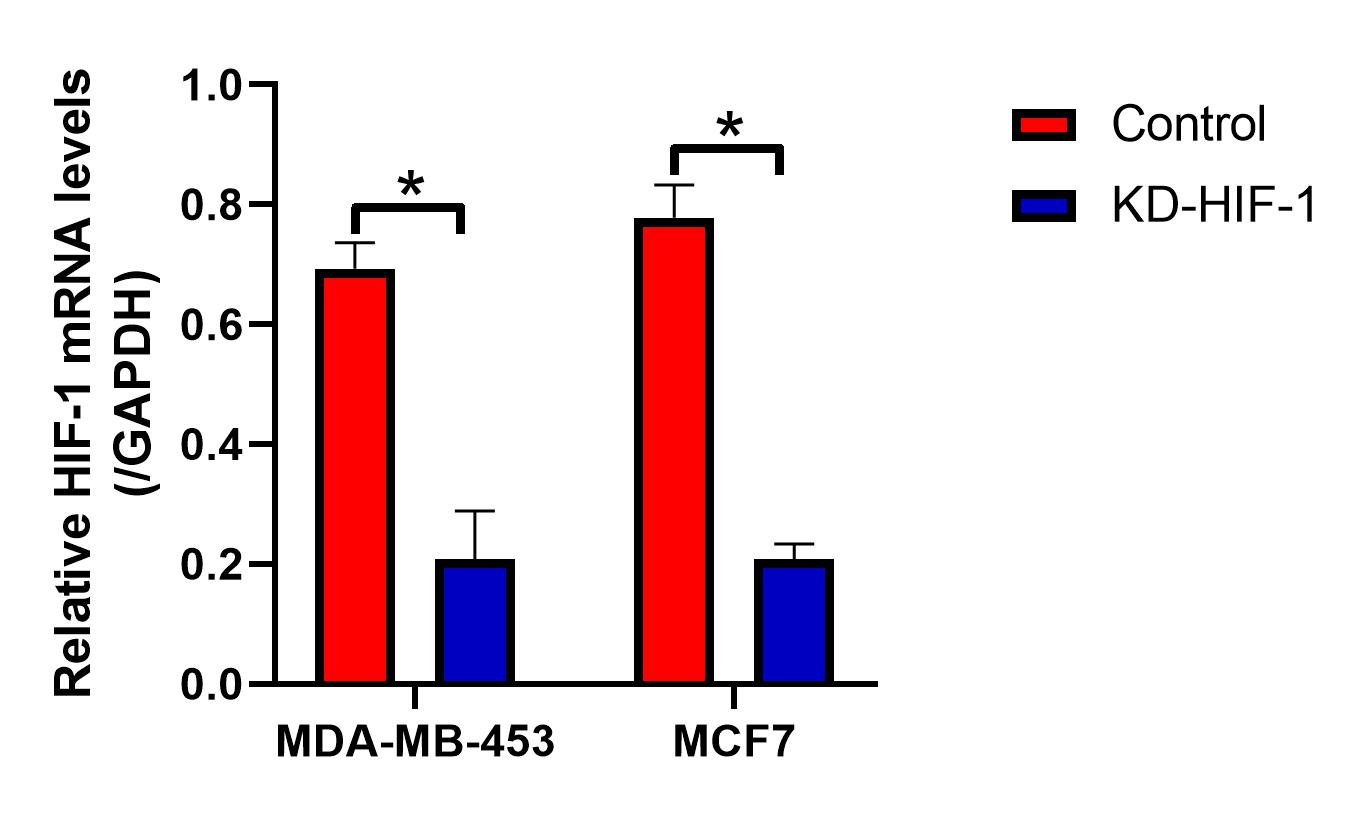


**Figure S6.** The transfection efficiency of HIF-1 knockdown vectors was determined by performing Real-Time qPCR analysis. **P* < 0.05
