# Supplementary material for: Hypoxia-Induced Intracellular and Extracellular Heat Shock Protein gp96 Increases Paclitaxel-Resistance and Facilitates Immune Evasion in Breast Cancer
Source: Front Oncol. 2021 Dec 20;11:784777. doi: 10.3389/fonc.2021.784777 (PMC8722103; doi:10.3389/fonc.2021.784777)

**Uncropped Western Blot images**

Figure 1C

1. Gp96


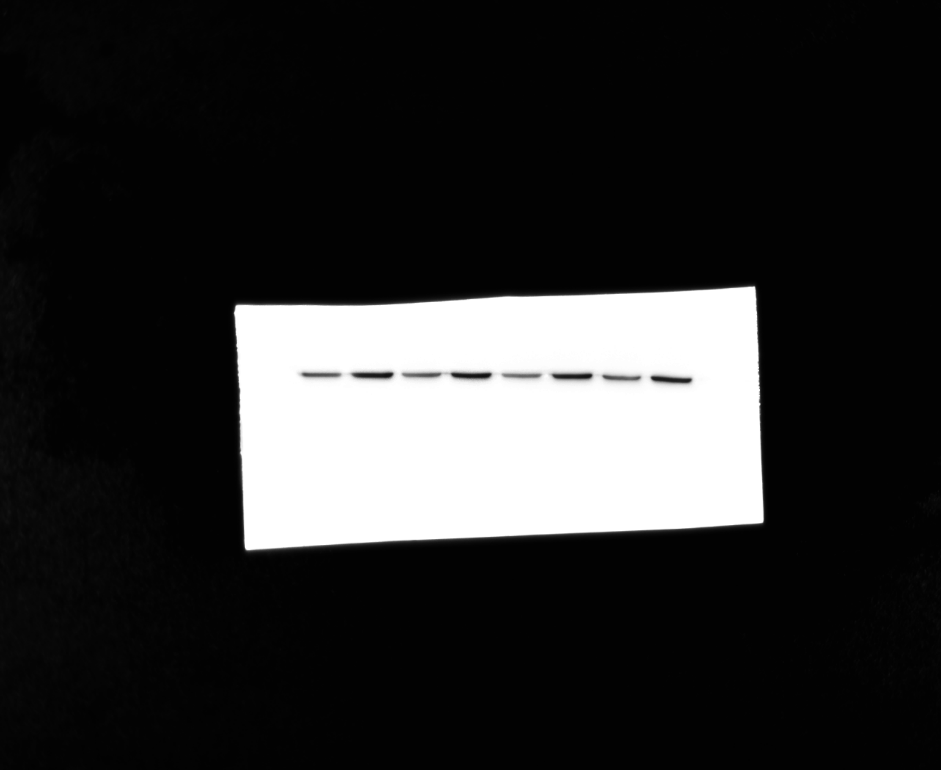


1. TSG101


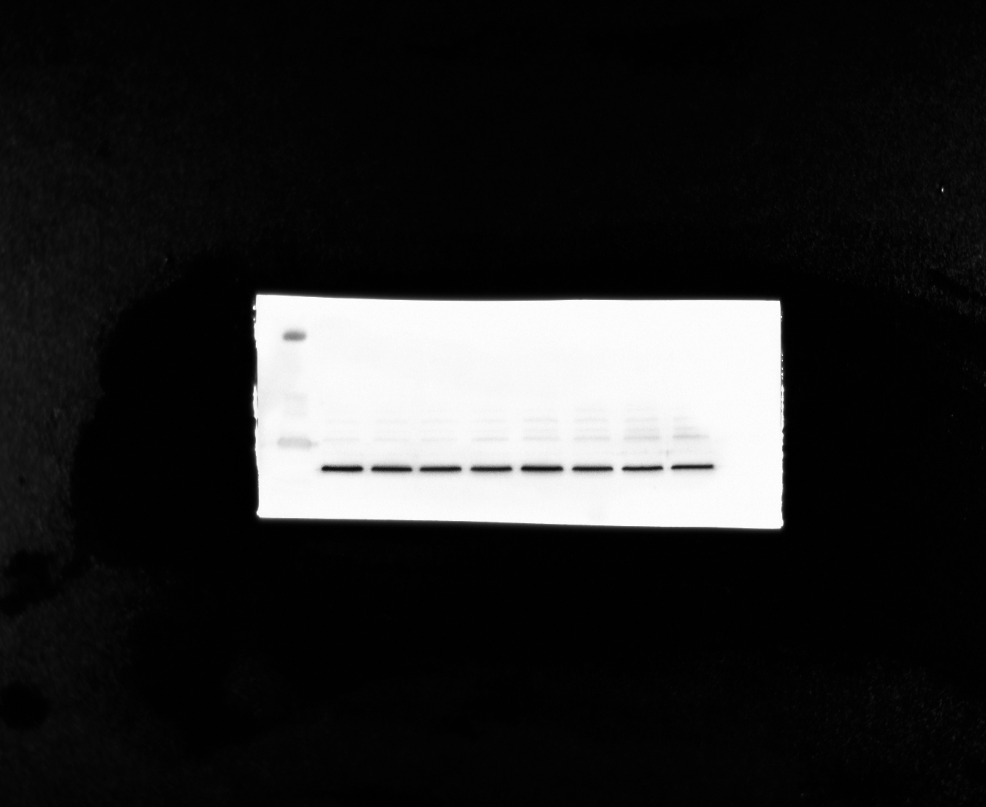


Figure 1K

1. Gp96


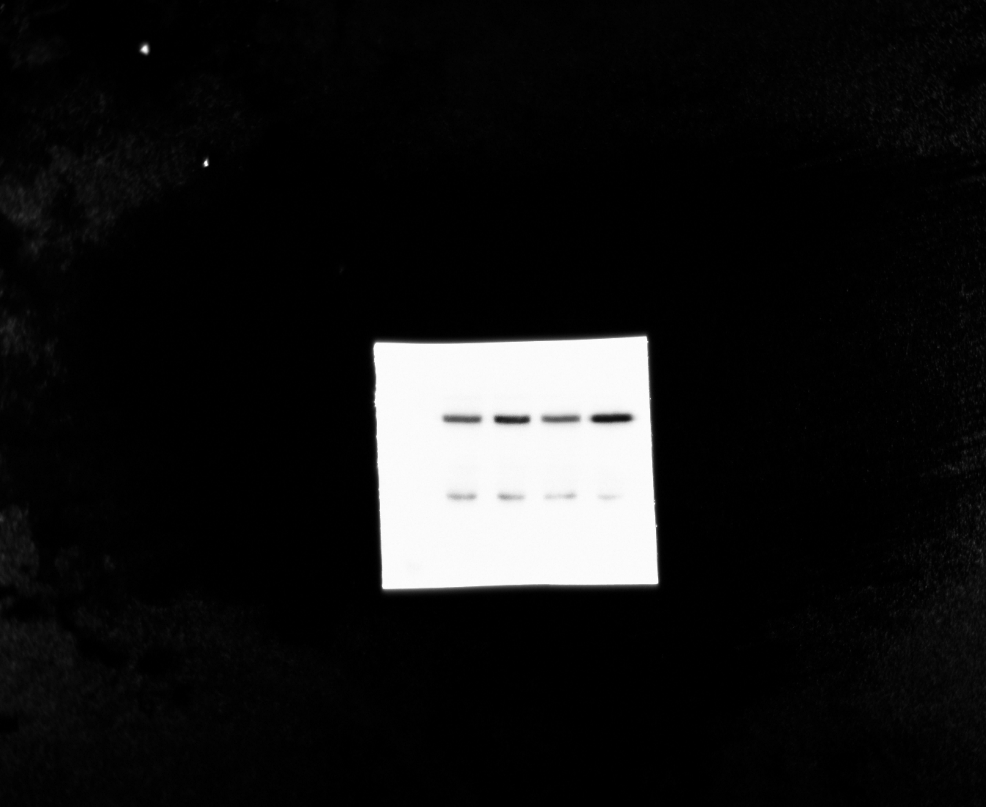


1. GAPDH


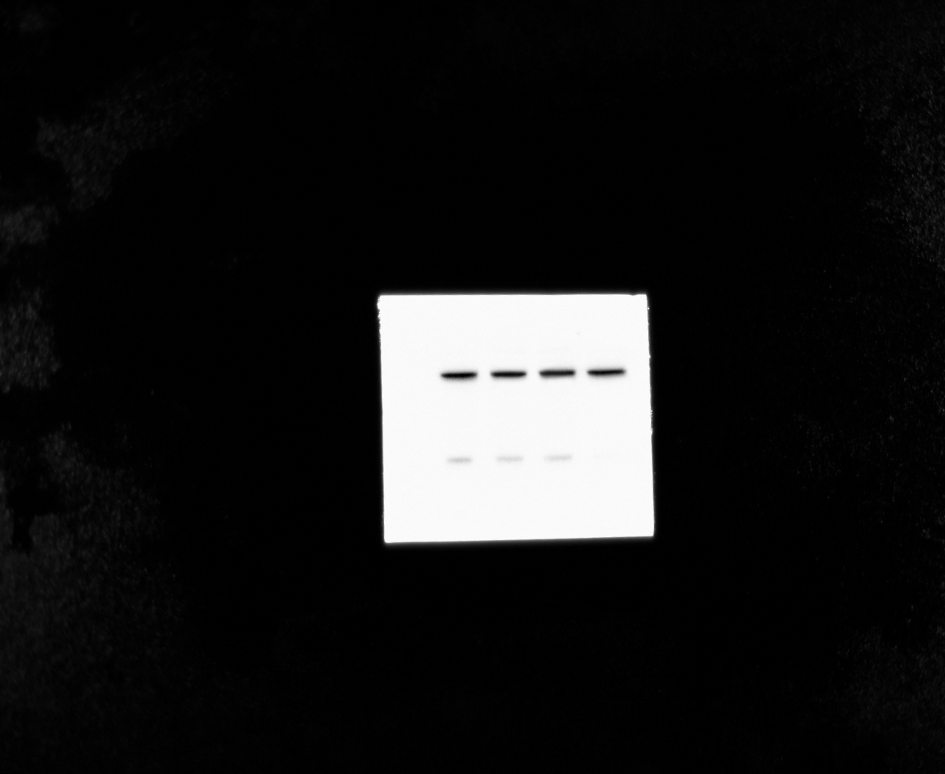


Figure 1M

1. Gp96


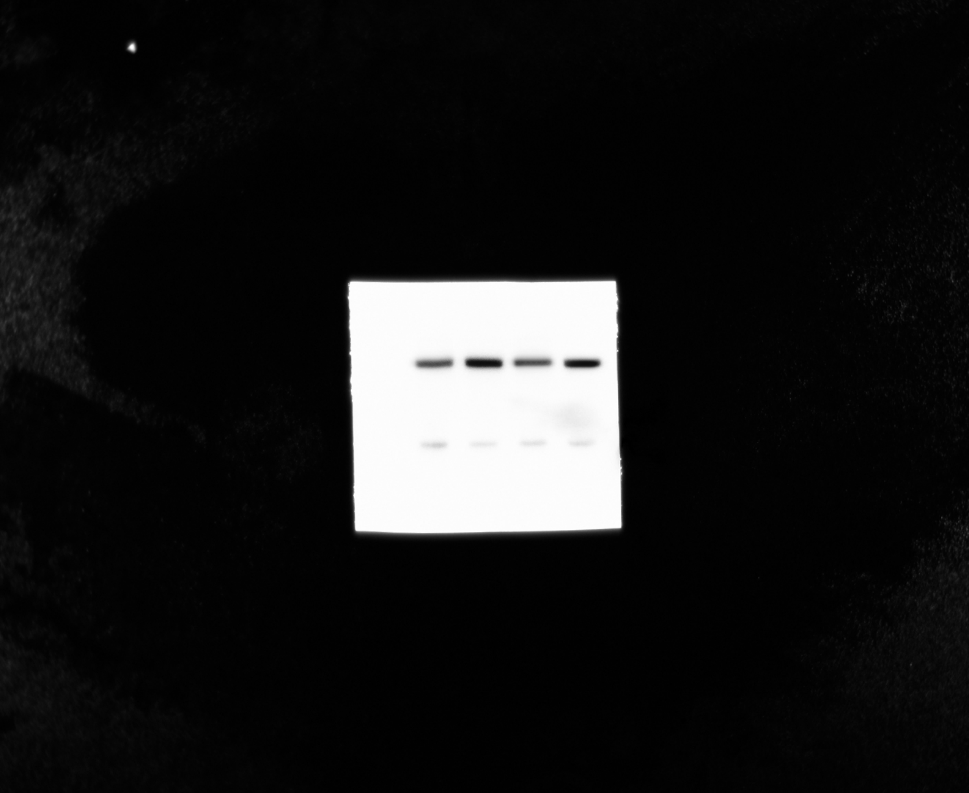


1. TSG101


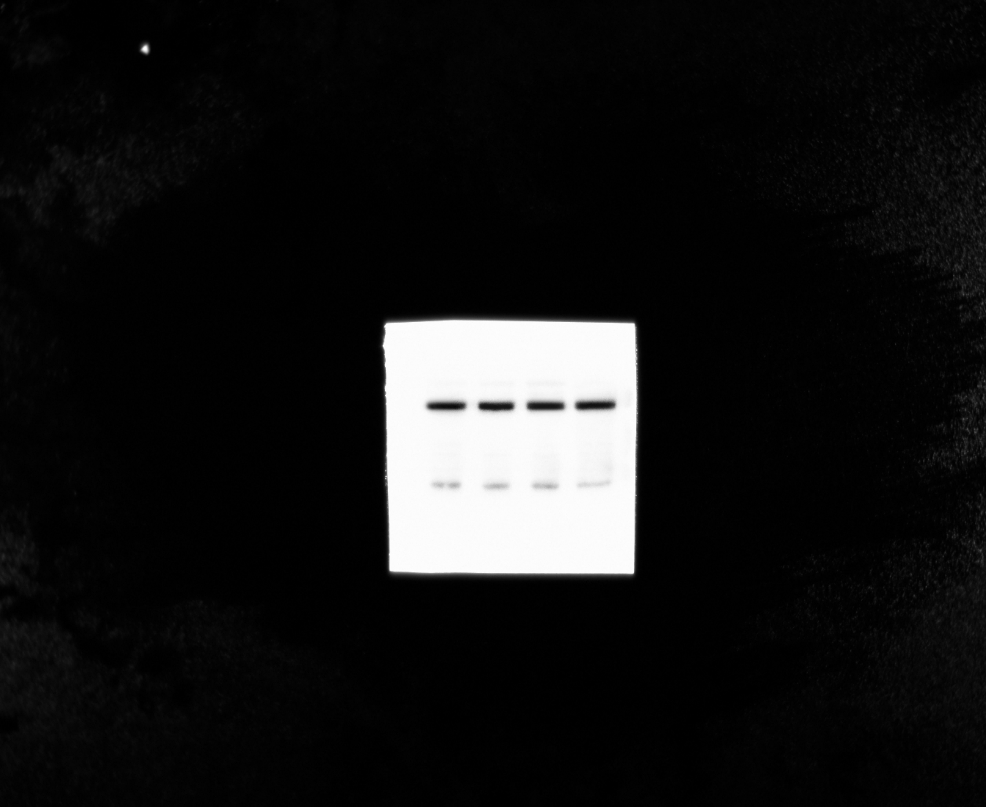


Figure 2H

1. P53


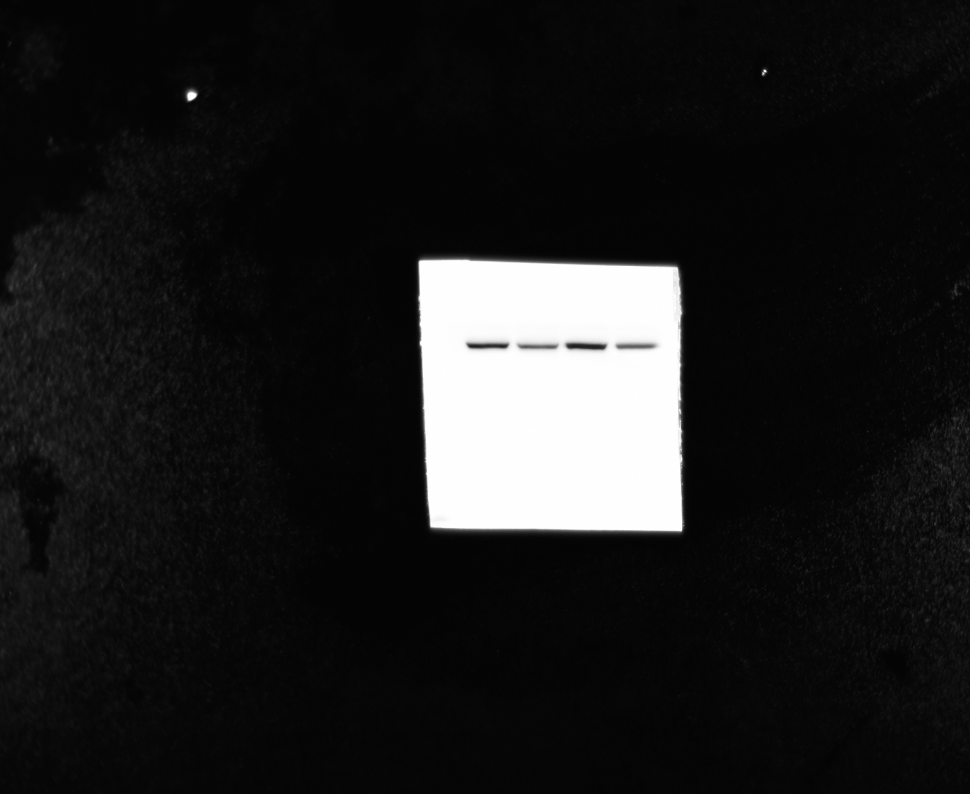


1. GAPDH


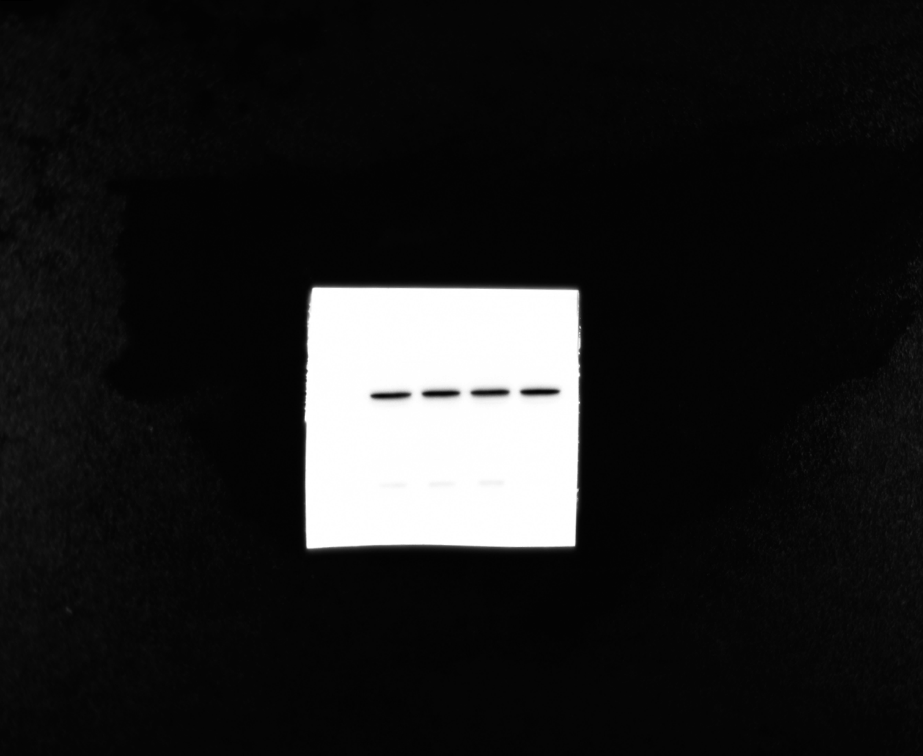


Figure 3B

1. Gp96


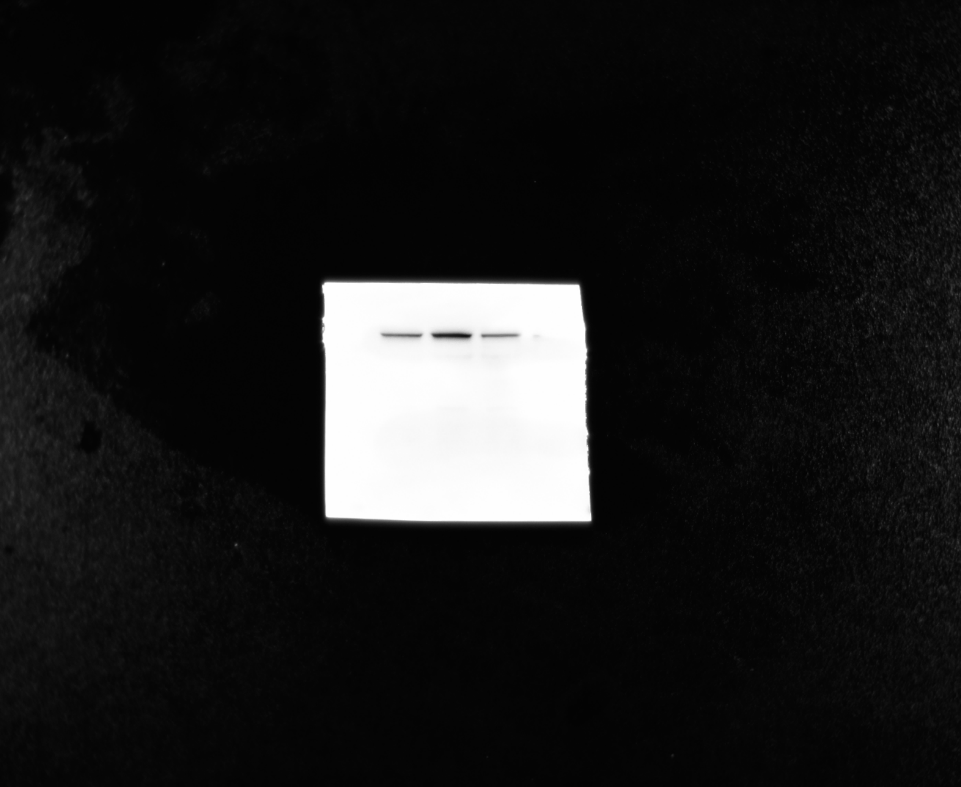


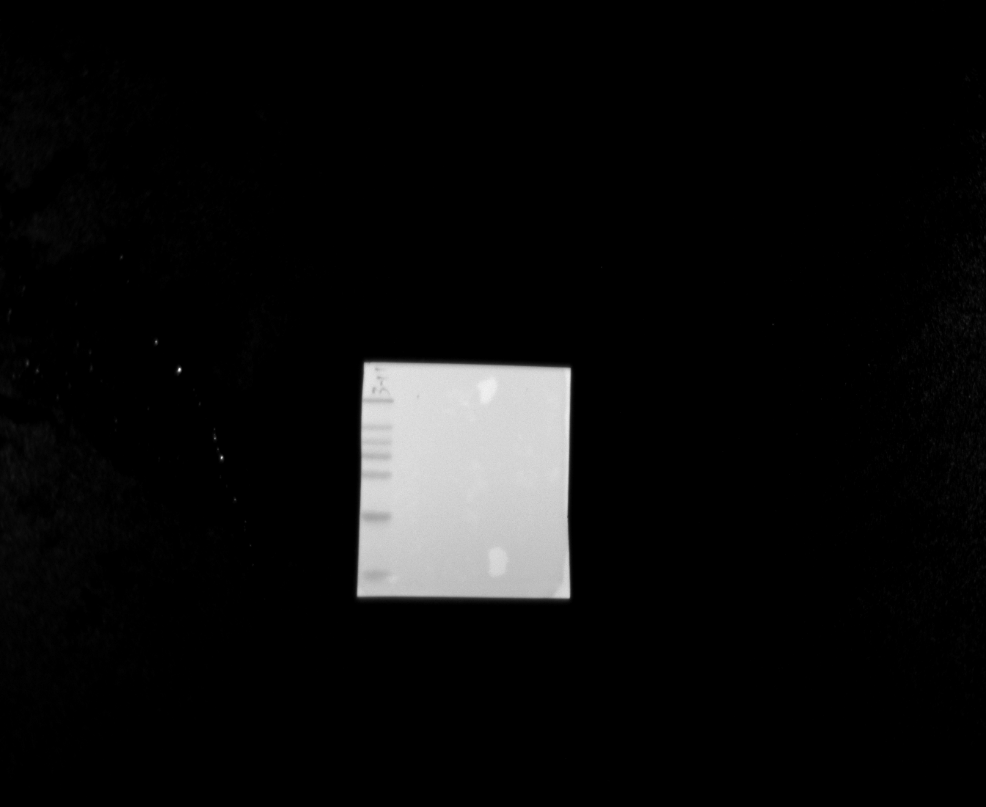


1. GAPDH


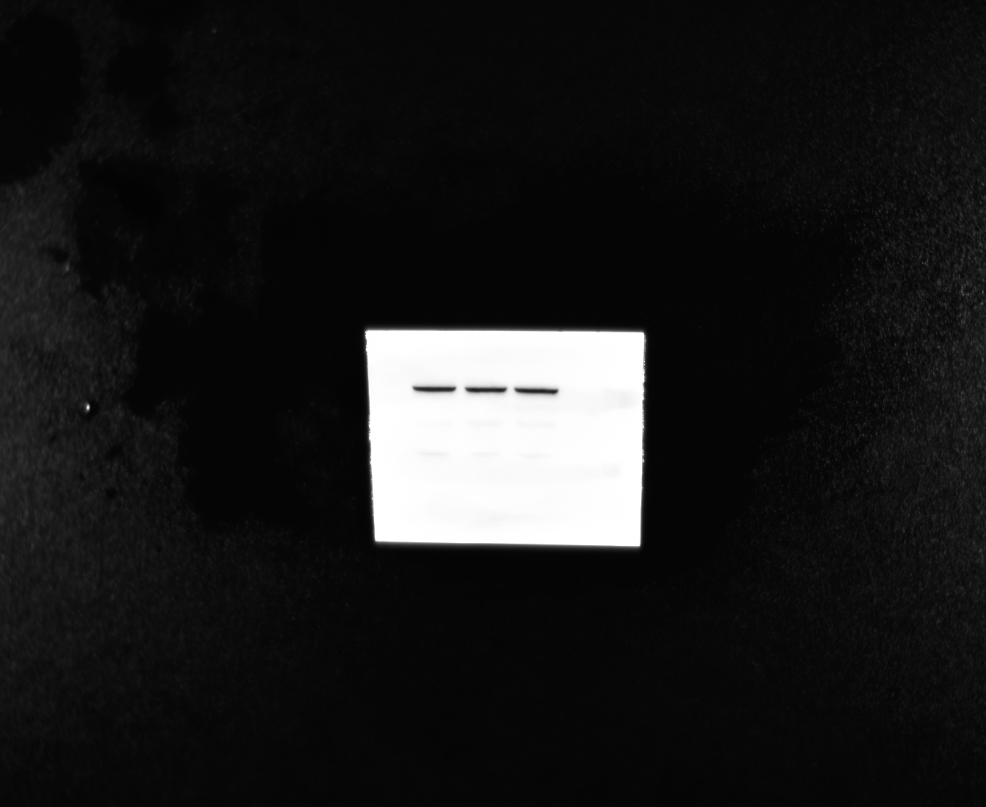


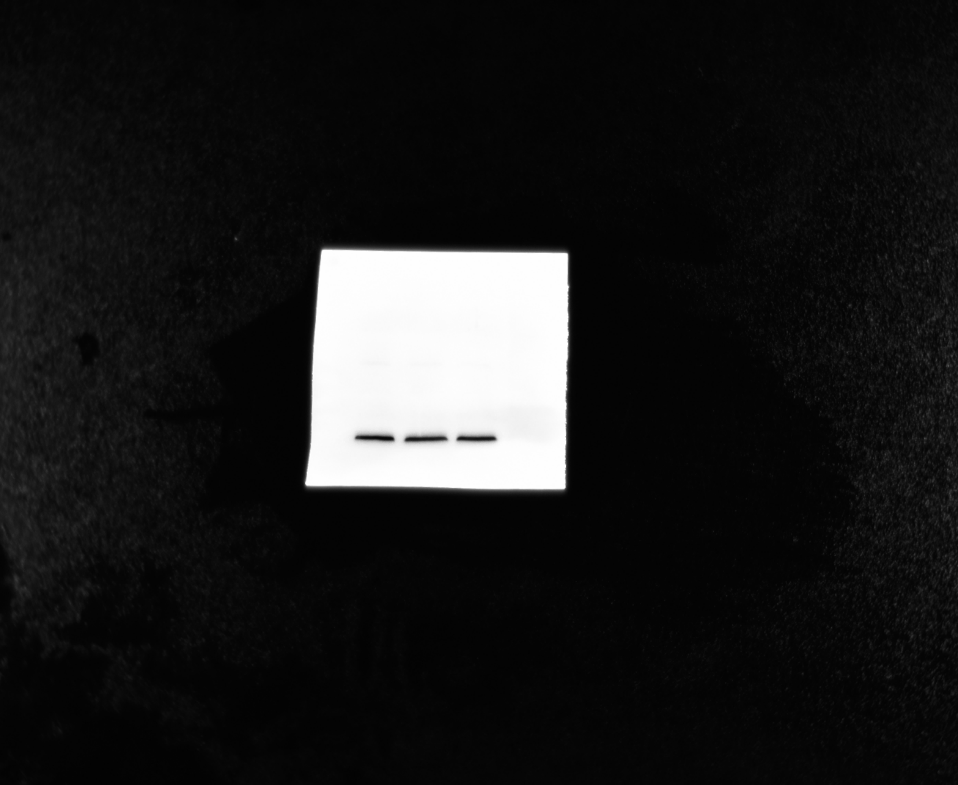


Figure 6B

1. Gp96


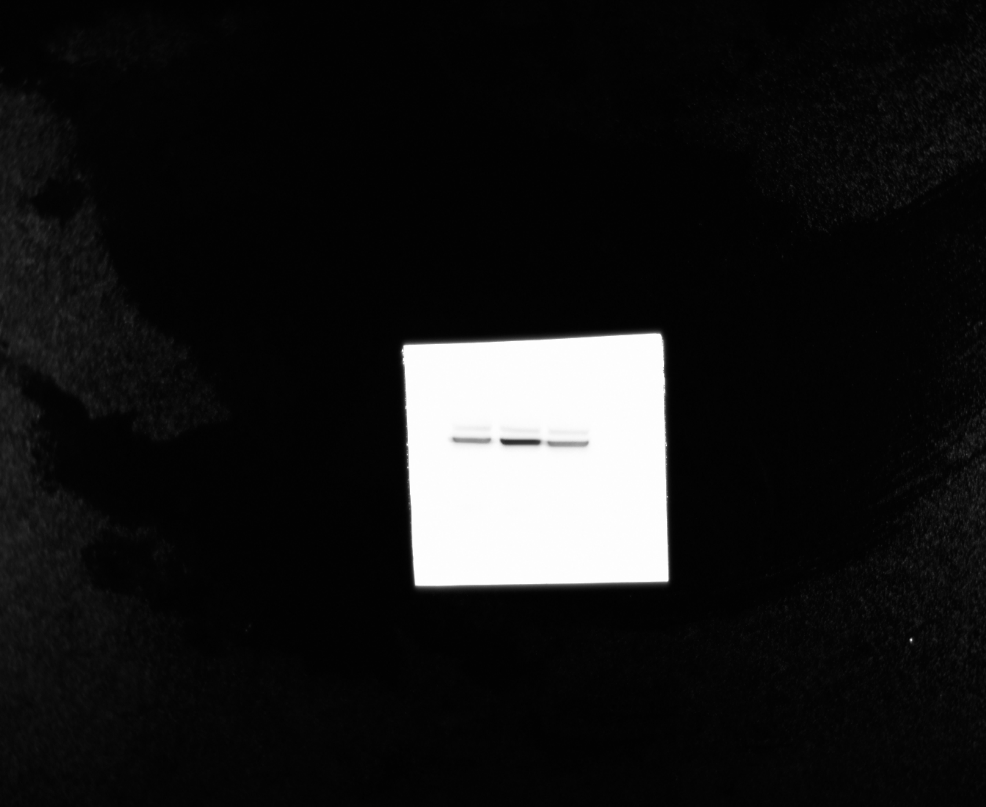


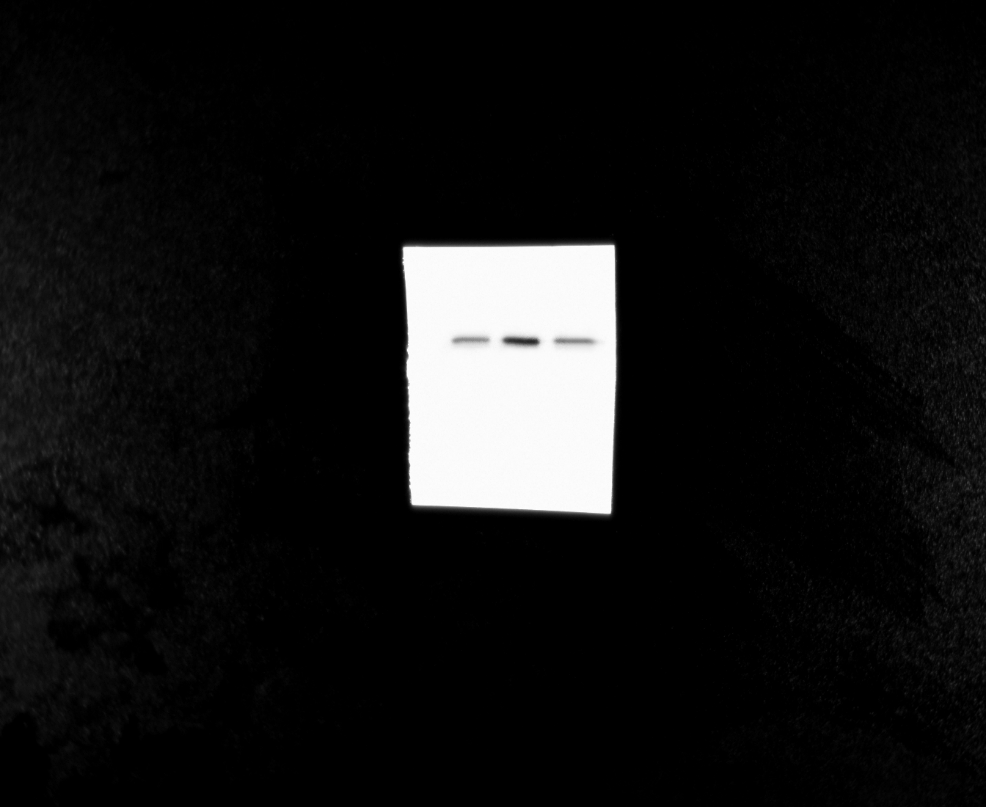


1. TSG101


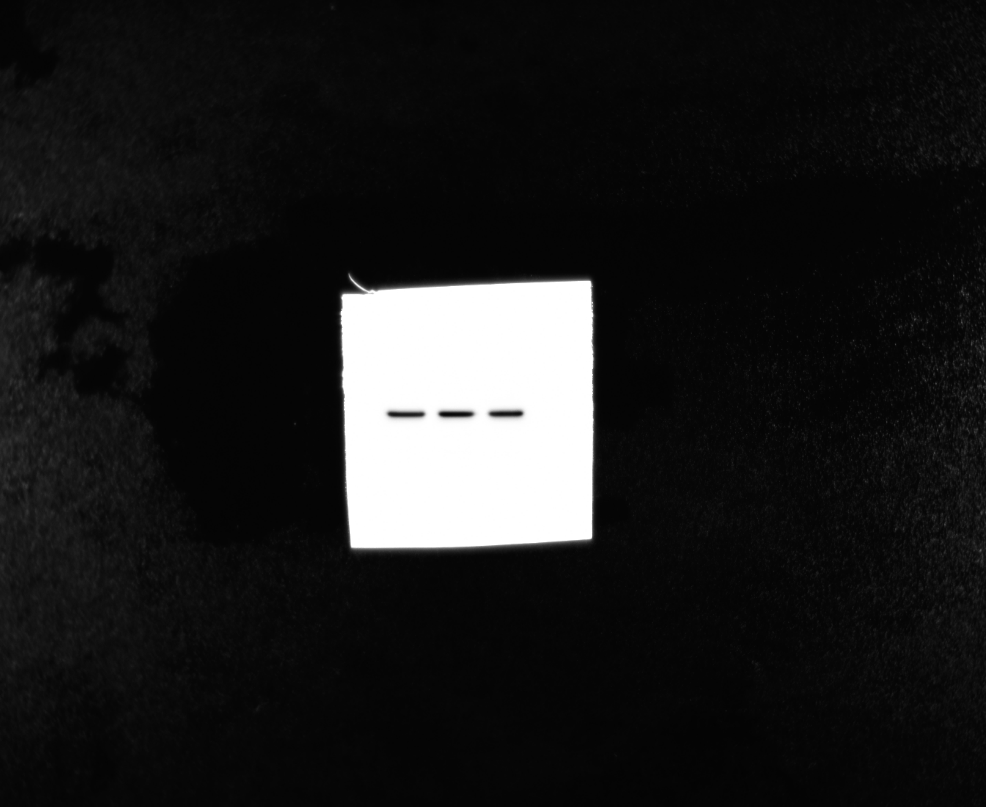


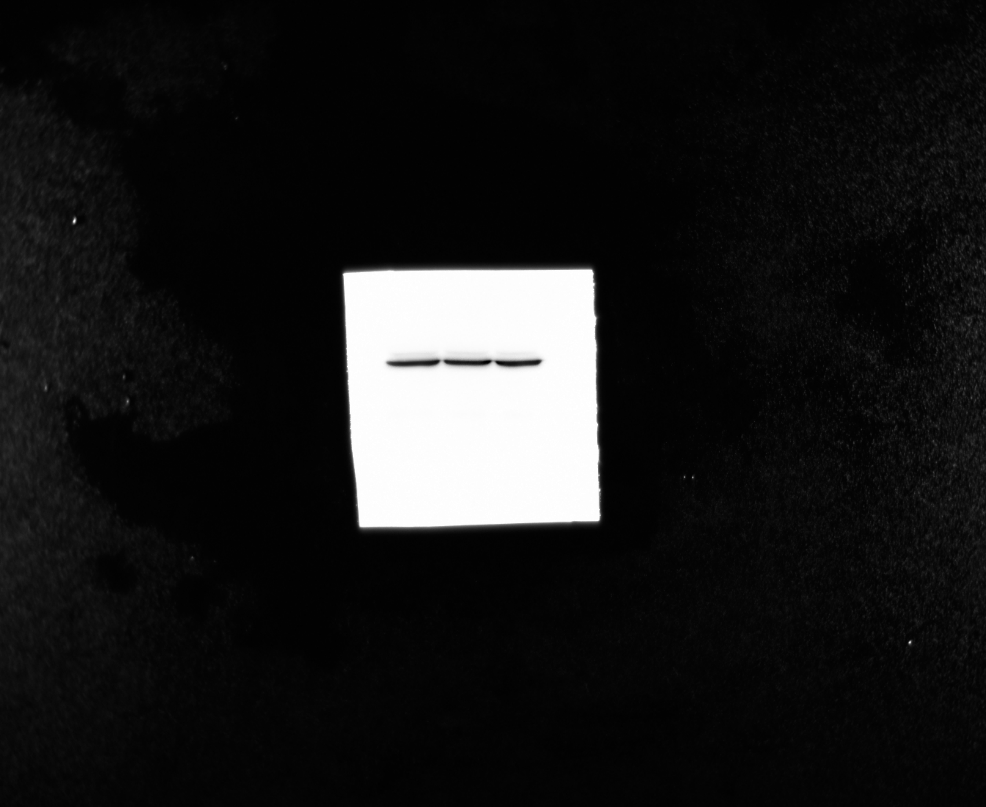


Figure S1

1. CD63


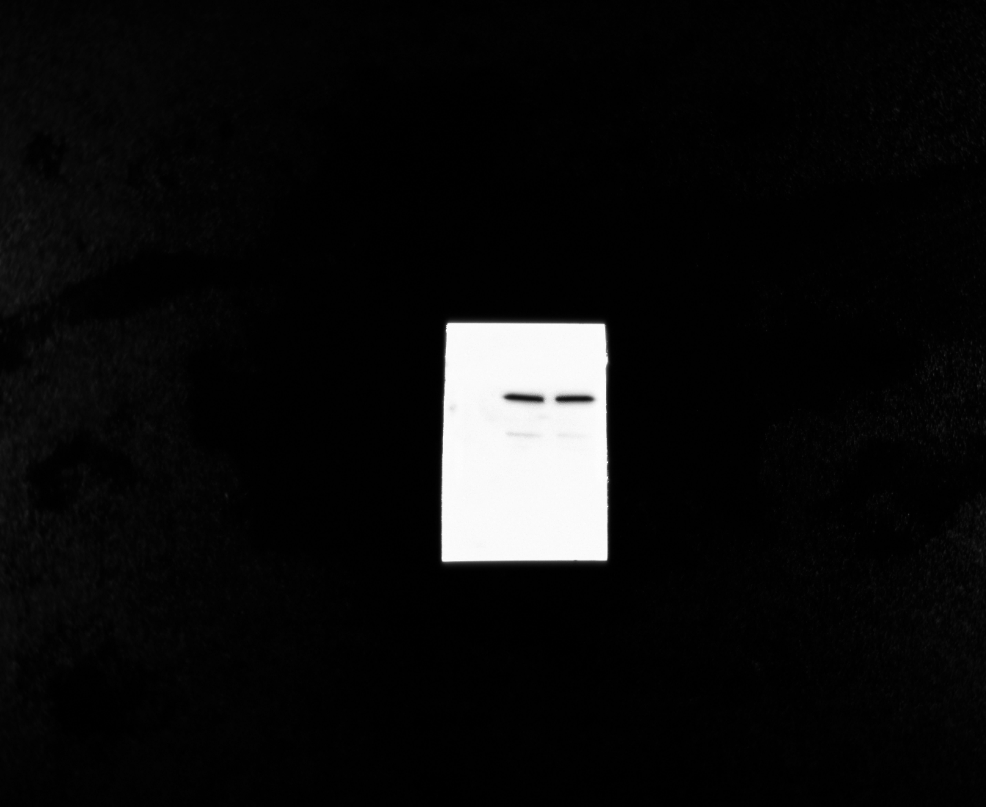


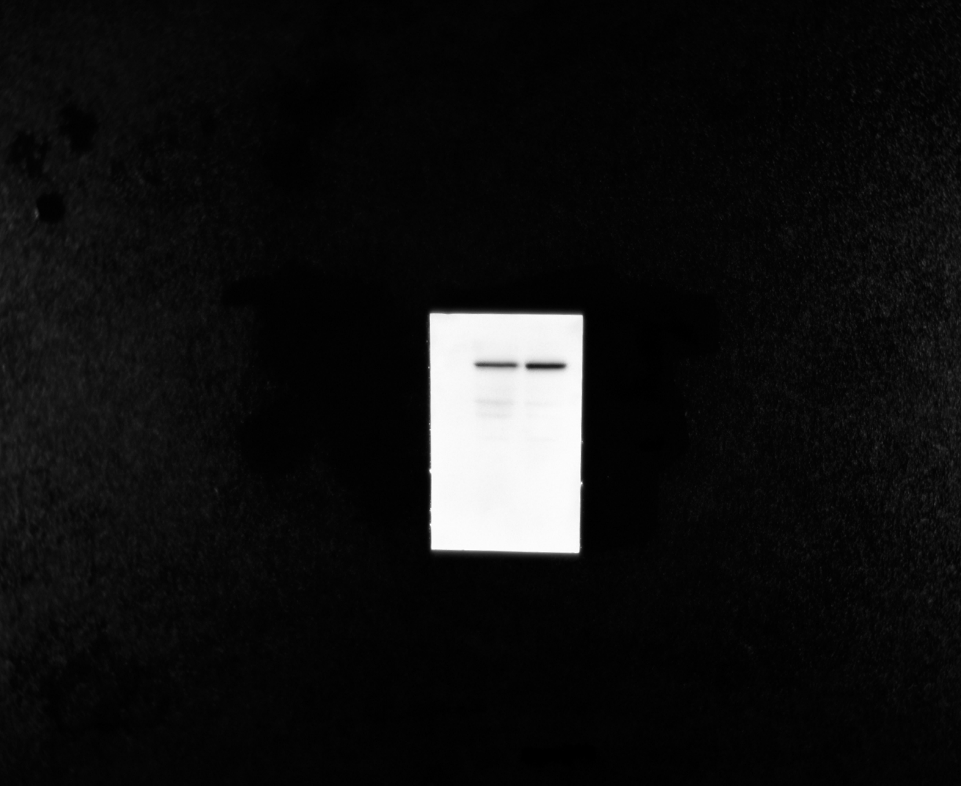


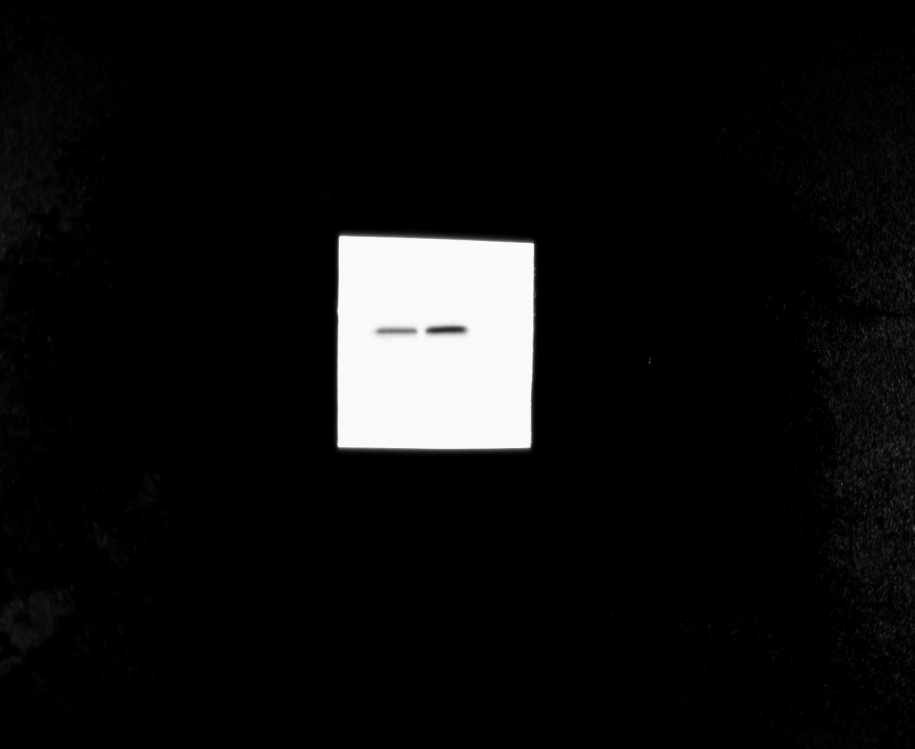


1. TSG101


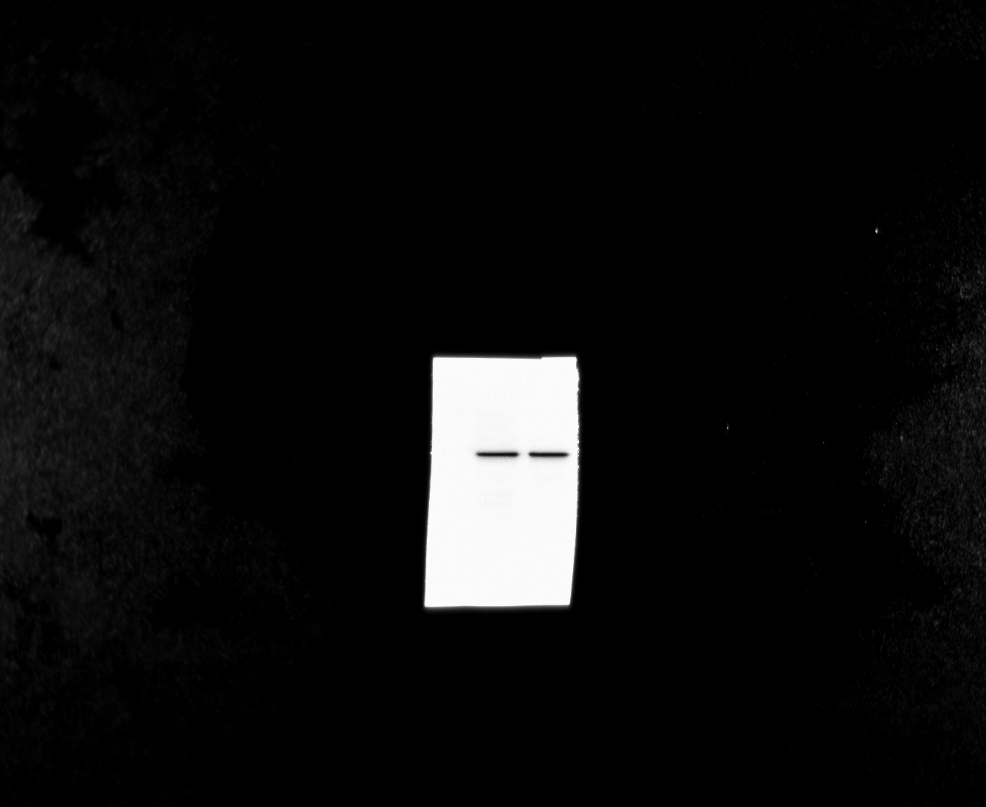


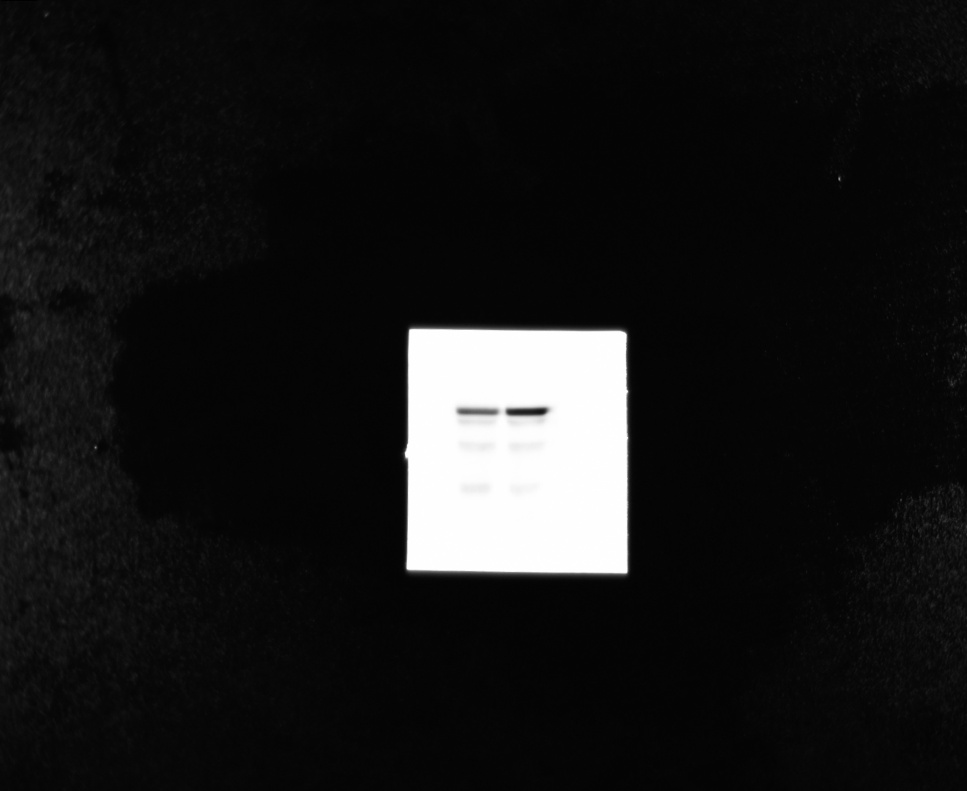


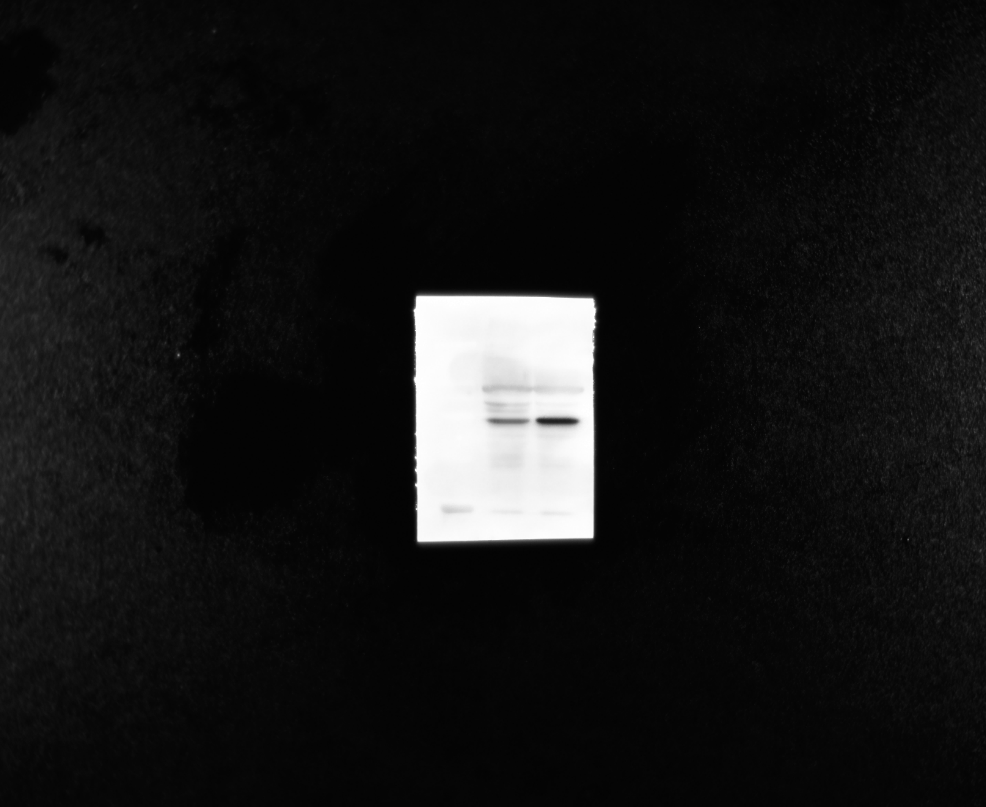


1. Actin


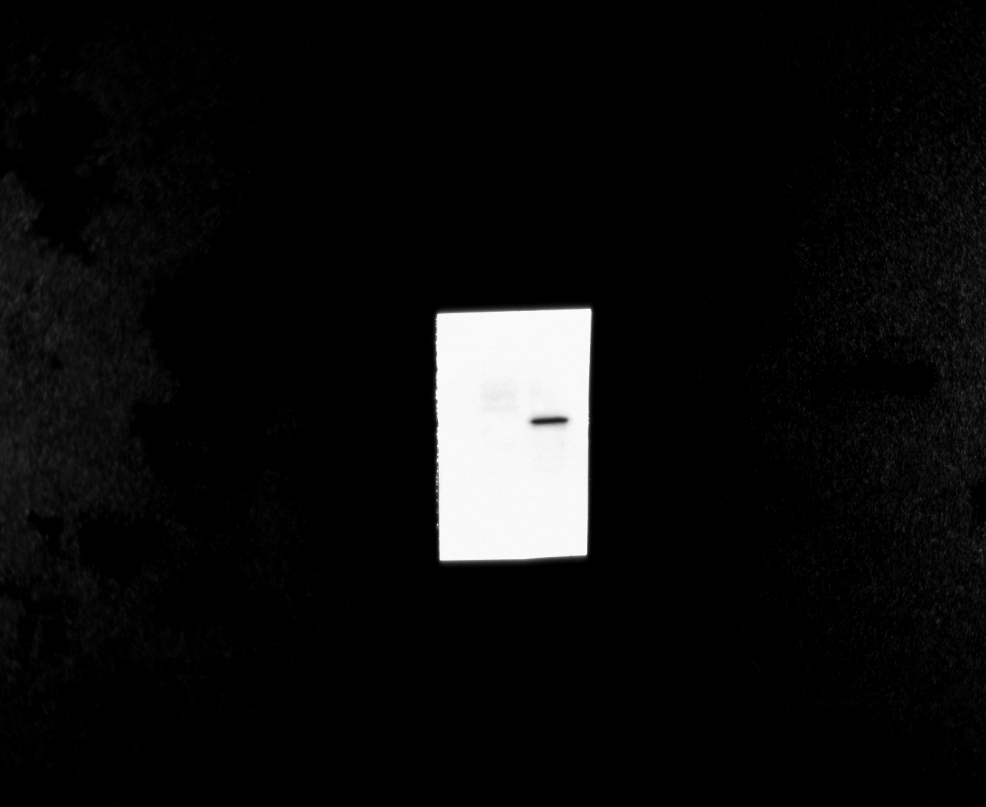


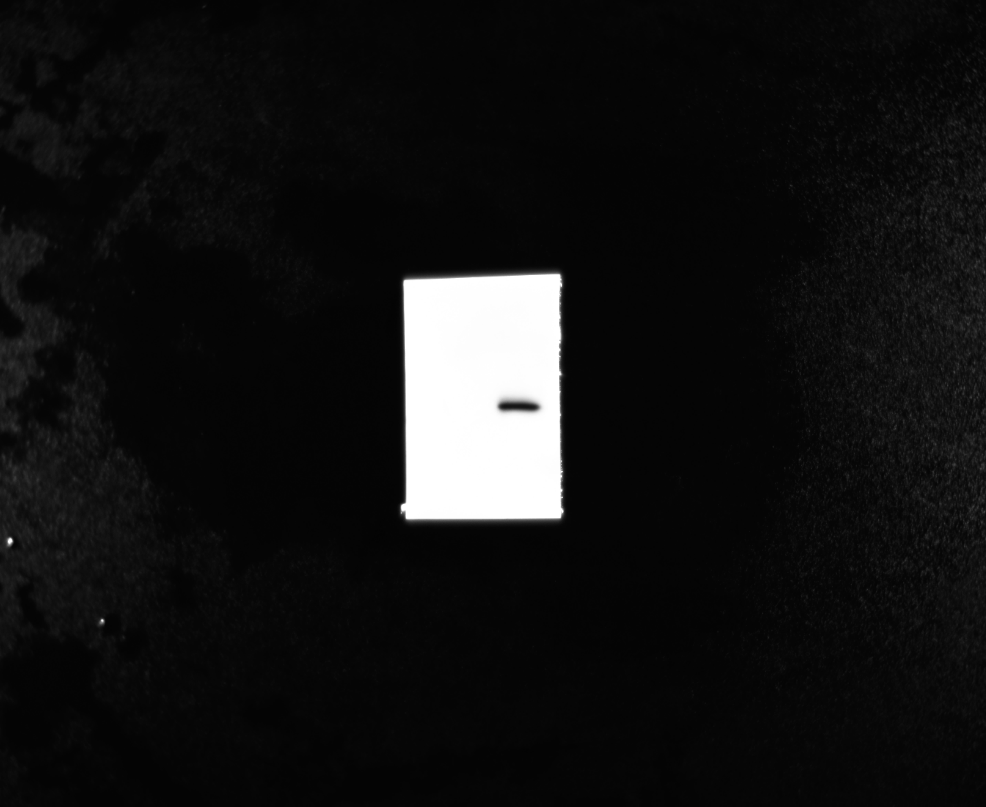


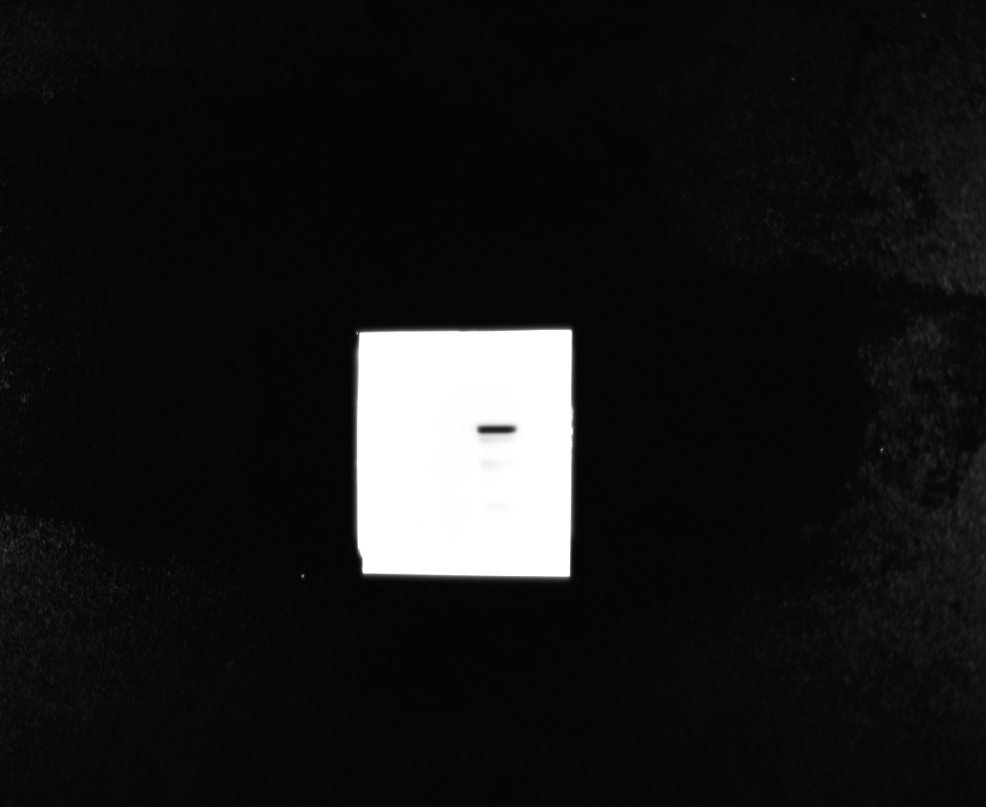


Figure S2D

1. p53


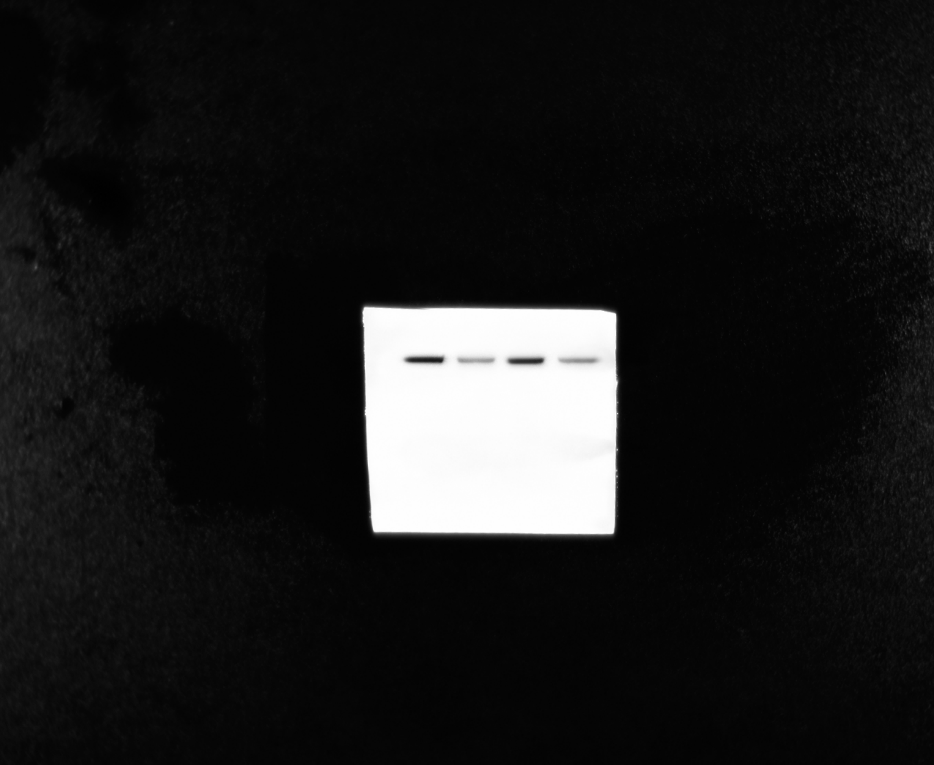


1. GAPDH


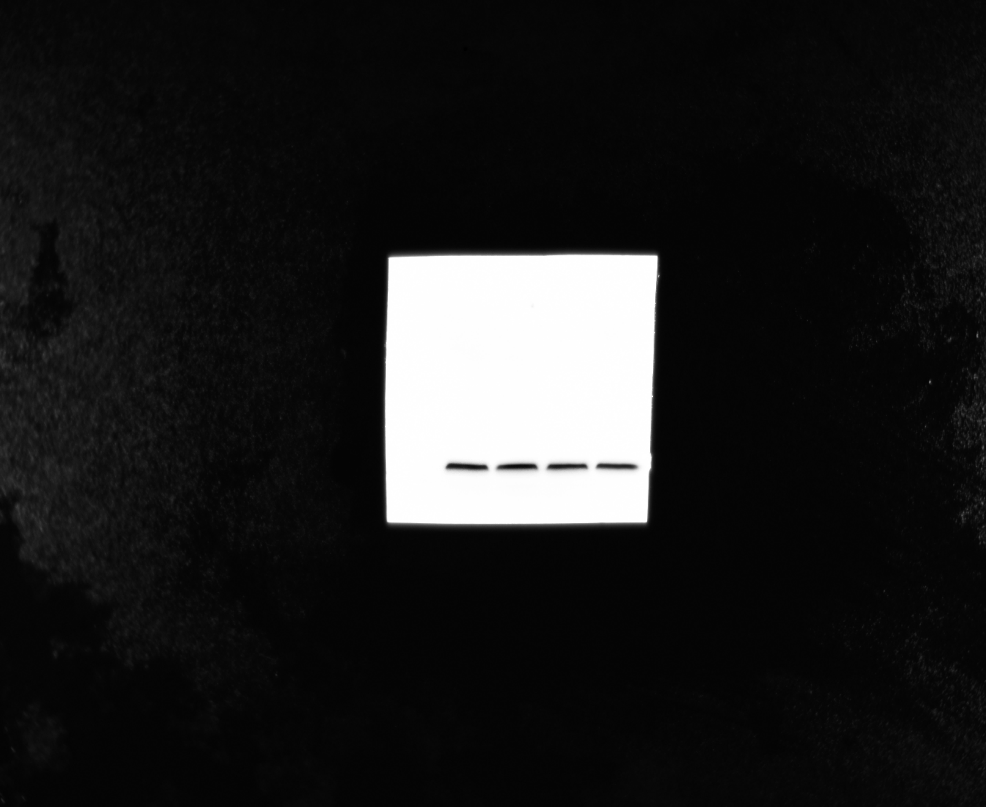

Supplement: Supplementary file 2 [file DataSheet_2.docx]
